# Supplementary material for: Nanopore-Based Direct RNA Sequencing of the Trypanosoma brucei Transcriptome Identifies Novel lncRNAs
Source: Genes (Basel). 2023 Feb 28;14(3):610. doi: 10.3390/genes14030610 (PMC10048164; doi:10.3390/genes14030610)
Supplement: Supplementary file 1 [file genes-14-00610-s001.zip › genes-2216228-supplementary.pdf]

Supplementary Material

Nanopore-based direct RNA sequencing of the *Trypanosoma brucei* transcriptome identifies novel lncRNAs

Elisabeth Kruse and H. Ulrich Göringer

Molecular Genetics, Technical University Darmstadt, Schnittspahnstraße 10, 64287 Darmstadt, Germany

Table of Contents

1. Supplementary Figures

**Figure S1**	Pairwise comparison of gene expression levels of all DRS libraries
**Figure S2**	Example for the distribution of transcript start- and stop-sites
**Figure S3**	Examples of SAS sites within annotated coding sequences
**Figure S4**	Side-by-side comparison of mapped SAS and PAS sites derived from DRS sequencing, Iluminna short-read seq., and Solexa short-read seq.
**Figure S5**	Representative examples of di-cistronic transcripts.
**Figure S6**	Chromosomal mapping of all newly identified lncRNA genes
**Figure S7**	Noncoding RNAs as precursors for snoRNAs
**Figure S8**	Sequence and structure comparison of coding and noncoding *T. brucei* transcripts
**Figure S9**	Complex clustering of lncRNA genes
**Figure S10**	Genomic context and differential expression of 4 newly identified *T. brucei* lncRNAs
**Figure S11**	Transcript coverage and A-nt content of the variable region of the *T. brucei* mitochondrial (maxicircle) genome
**Figure S12**	DRS-derived steady-state levels of mitochondrial transcripts in procyclic-stage and bloodstream-stage *T. brucei*
**Figure S13**	Polyadenylation analysis of *T. brucei* mitochondrial RNAs

2. Supplementary Tables

**Table S1**	Read statistics of DRS sequencing data
**Table S2**	Transcript mapping statistics
**Table S3**	Identification of full-length sequencing reads
**Table S4**	Performance of lncRNA prediction tools
**Table S5**	Genomic locations of novel intergenic lncRNAs
**Table S6**	Differential gene expression. Genes up-regulated in bloodstream-stage *T. brucei*
**Table S7**	Differential gene expression. Genes up-regulated in procyclic-stage *T. brucei*

1. Supplementary Figures

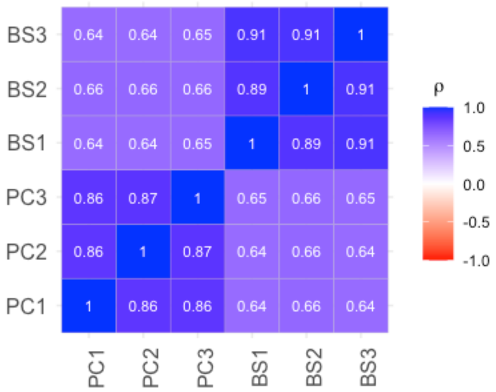

Supplementary Figure S1. Pairwise comparison of gene expression levels of all DRS sequencing libraries. BS1, BS2, BS3: libraries from bloodstream-stage *T. brucei*. PC1, PC2, PC3: libraries from procyclic-stage parasites. Gene expression levels were determined using featureCounts from the R-package Rsubread [38]. *p*=Spearman rank correlation coefficient. Exons with low expression levels (<6 reads) were not considered.

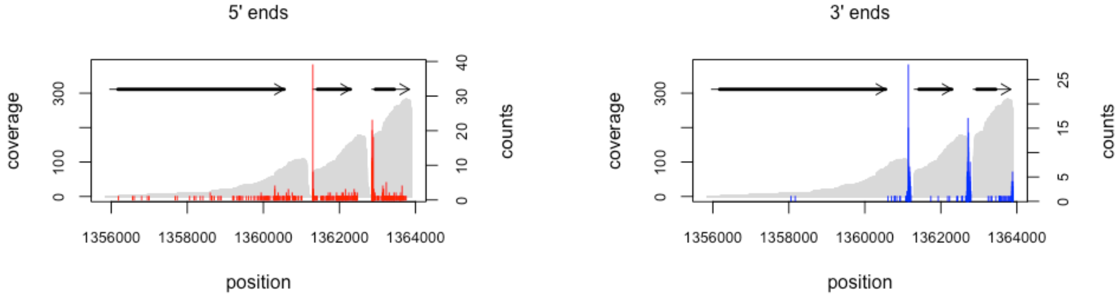

Supplementary Figure S2. Distribution of transcript start- and end-positions. Coverage profiles (grey) from bloodstream-stage trypanosomes for the genomic region Tb927_07_v5.1:1356000-13764000. Transcript start sites are in red and transcript end-positions are in blue. Grey arrows are annotated exons. Bold, black lines are coding sequences.

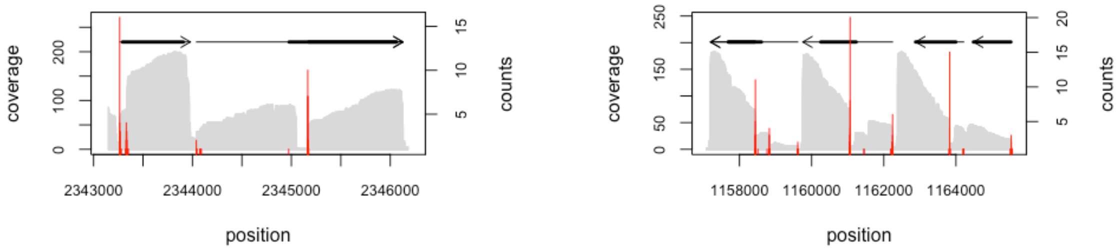

Supplementary Figure S3. Examples of splice acceptor (SAS) sites located within annotated coding sequences. Coverage profiles (grey) of two representative regions in the *T. brucei* genome (Tb927_09_v5.1:2343000-2346000(+), Tb927_08_v5.1:1157080-1165560(-), derived from bloodstream-stage trypanosomes. SAS sites, identified by the presence of a 5'-spliced leader (SL)-sequence are shown in red. Peak heights indicate read numbers. Grey arrows are annotated exons. Bold black lines are coding sequences.

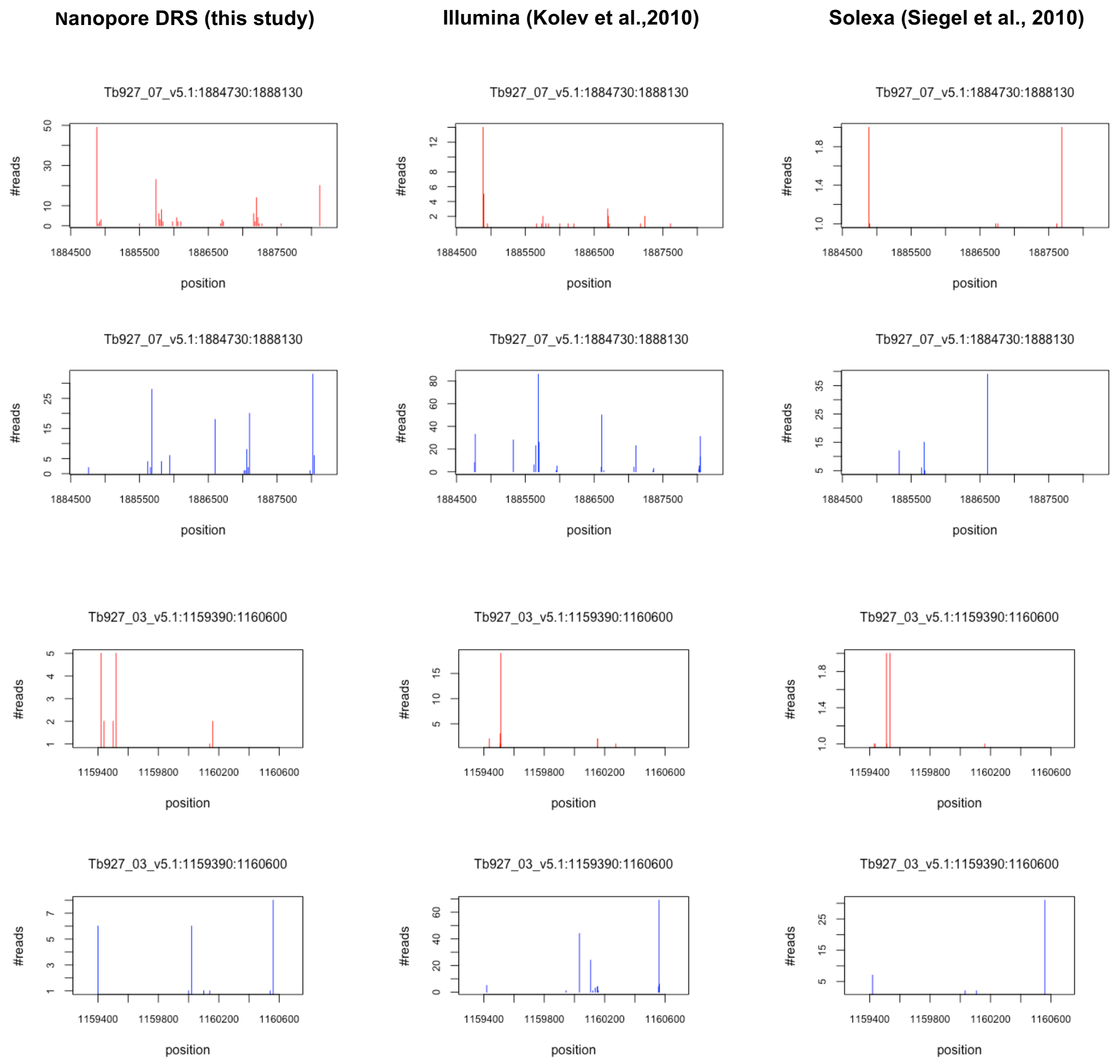

Supplementary Figure S4. Side-by-side comparison of mapped splice acceptor (SAS, red) and polyadenylation (PAS, blue) sites for two selected regions of the *T. brucei* genome, derived from different sequencing methods. Left: Nanopore-based DRS (this study). Center: Short-read Illumina sequencing [15]. Right: Short-read Solexa sequencing [48]. For the DRS data, the number of reads (start- or end-positions) was summed up over a window of 20nt.

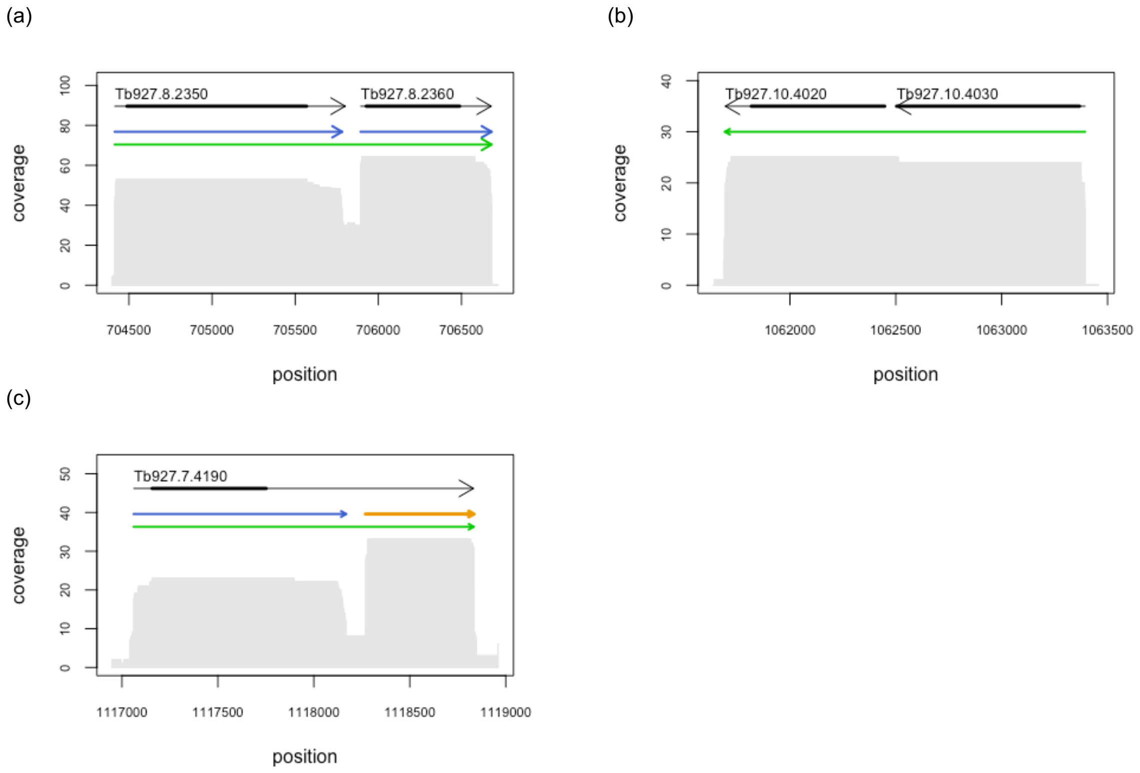

Supplementary Figure S5. Representative examples of di-cistronic transcripts. (a) Coverage profile of a di-cistronic transcript (green arrow) involving the *T. brucei* genes Tb927.8.2350 and Tb927.8. 2360. Blue arrows represent the monocistronic RNAs. (b) Example of a misannotation involving the annotated loci Tb927.10.4020 and Tb927.10.4030. Only a single transcript was identified (green arrow). (c) Coverage profile of a di-cistronic transcript (green arrow) encoding gene Tb927.7.4190 and a 3'-UTR-located lncRNA (arrow in orange). The monocistronic transcript is shown as a blue arrow. Grey arrows are annotated exons. Bold black lines are coding sequences. Coverage profiles are from full-length reads.

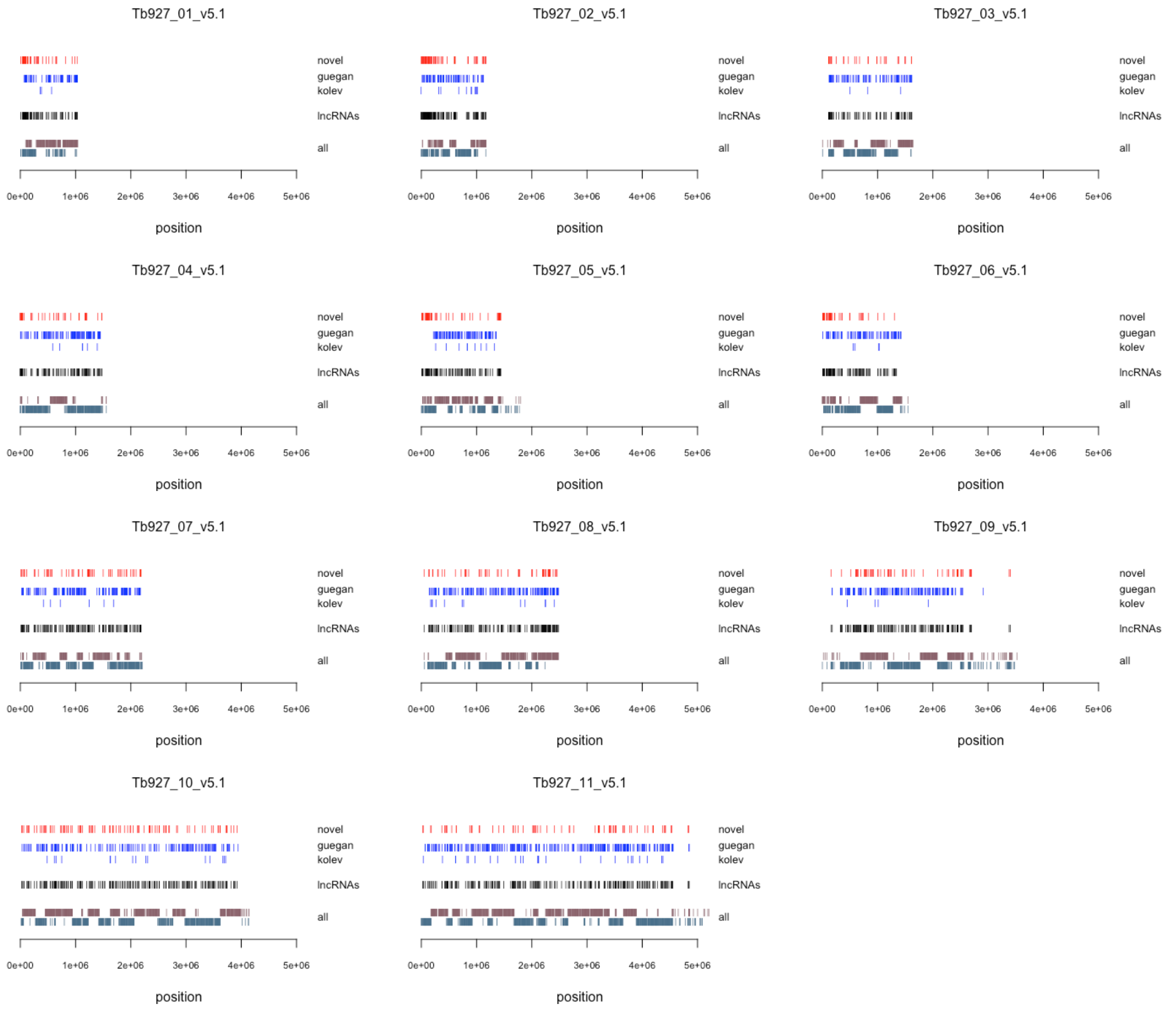

Supplementary Figure S6. Localization of all newly identified lncRNA genes (this study, red) on the 11 Mbp-size chromosomes of *T. brucei* (top to bottom) in comparison to the published sequences by Guegan et al., 2022 [16] (blue) and Kolev et al., 2010 [15] (grey). Black: all three datasets combined. All: strand-specific representation of all lncRNA genes. Top: plus strand. Bottom: minus strand.

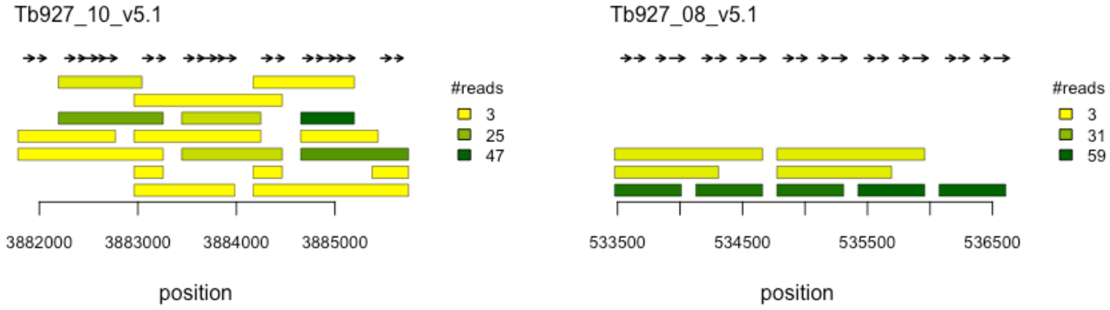

Supplementary Figure S7. Noncoding RNAs as precursors of snoRNAs. Examples from two regions of the *T. brucei* genome on chromosome 10 (left) and chromosome 8 (right). Horizontal bars are lncRNAs. Colors indicate the number of reads as indicated. Annotated snoRNAs are shown as black arrows.

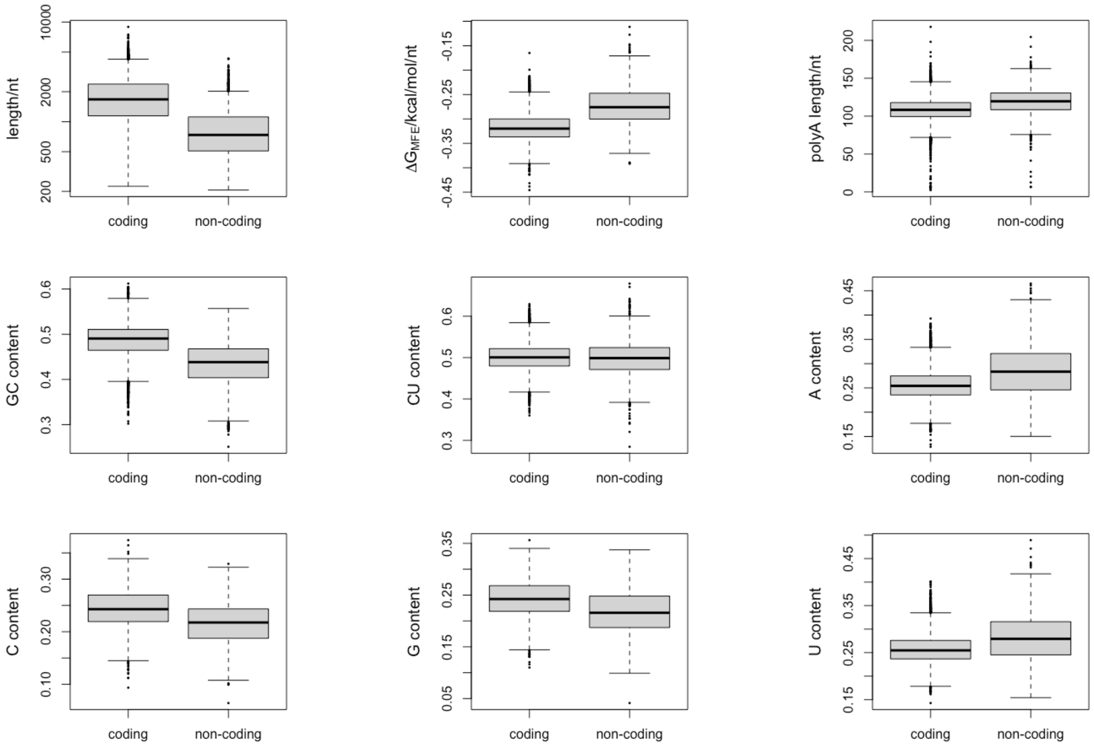

Supplementary Figure S8. Box-plot comparison of coding and noncoding transcripts from *T. brucei* for sequence and 2D-structure-specific parameters including nucleotide (nt) length, nt-content (A, C, G, U), GC- and CU-content, poly(A)-tail length, and thermodynamic stability (ΔG) of the minimal free energy structure (MFE). Transcripts were identified based on full-length reads as detailed in the Materials and Methods section. The coding potential was assessed by CPC2 [36] and LncFinder [37]. Only transcripts predicted as noncoding by both software tools were considered. ΔG-calculations were performed using RNAfold. Poly(A)-tails were assessed with the help of nanopolish [33].

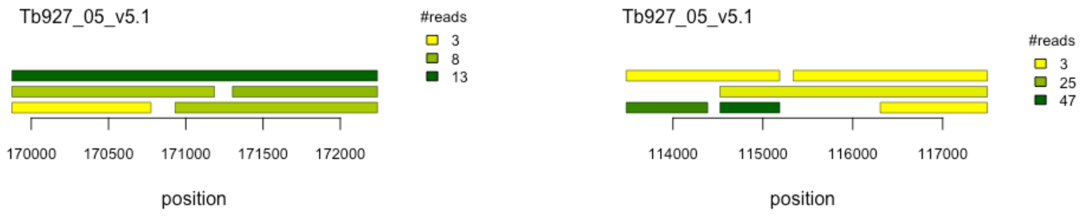

Supplementary Figure S9. Complex clustering of lncRNA genes. Examples from two different regions on chromosome 5 of the *T. brucei* genome are shown. Horizontal bars represent lncRNAs with colors indicating the number of sequencing reads.

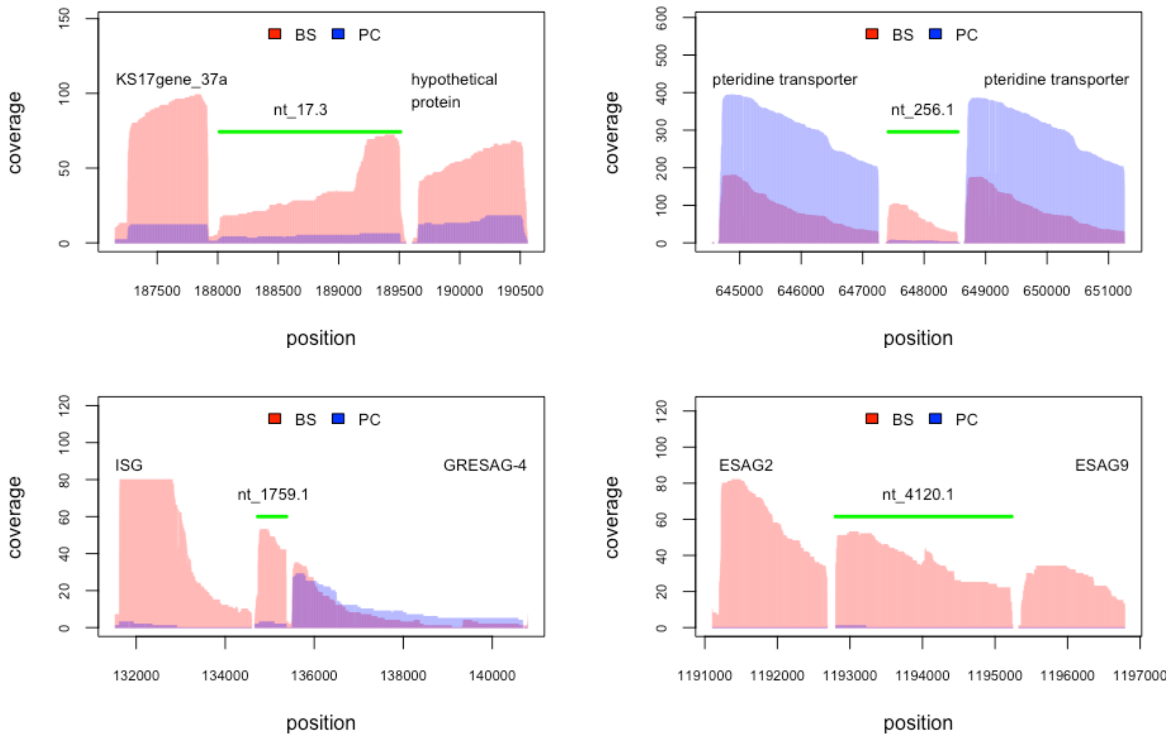

Supplementary Figure S10. Genomic context and differential expression of 4 newly identified lncRNAs (nt_17.3, nt_256.1, nt_1759.1, nt_4120.1) shown as green lines. Overlay plots of sequencing coverage profiles from bloodstream-stage (BS, red) and procyclic-stage (PC, blue) trypanosomes are shown. Flanking genes include the lncRNA gene K1717gene_37a, pteridine transporter genes, an invariant surface glycoprotein (ISG), the expression site-associated genes (ESAG) 2 and 9 and the glycine-rich expression site-associated gene 4 (GRESAG-4).

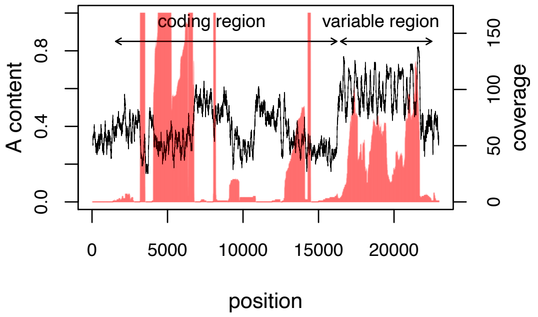

Supplementary Figure S11. Transcript coverage in the variable (noncoding) region of the *T. brucei* mitochondrial genome correlates with the local A-nucleotide content. Overlay plot of a DRS sequencing coverage profile (red) with the local A-nt content (black) of the plus strand of the mitochondrial genome. The data are derived from all procyclic-stage DRS libraries and the A-nt content was calculated over window of 100nt.

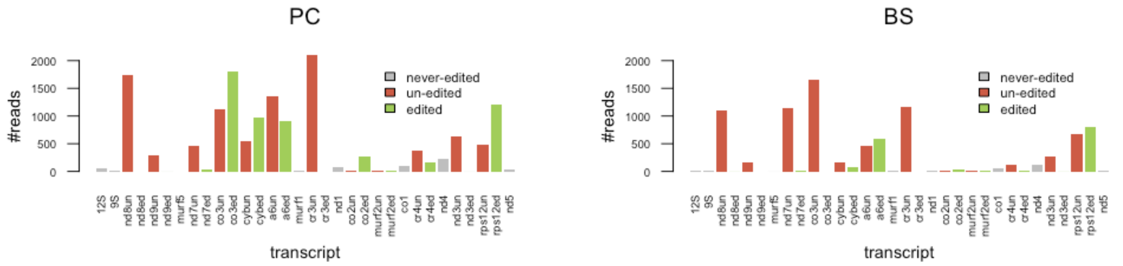

Figure S12. Steady-state levels of mitochondrial transcripts in procyclic-stage (PC) and bloodstream-stage (BS) *T. brucei*. Reads were mapped to the mitochondrial transcriptome and steady state RNA levels were estimated from the number of sequencing reads. Grey=never-edited transcripts. Red=un-edited RNAs. Green=edited RNAs. Gene annotations are as in [60].

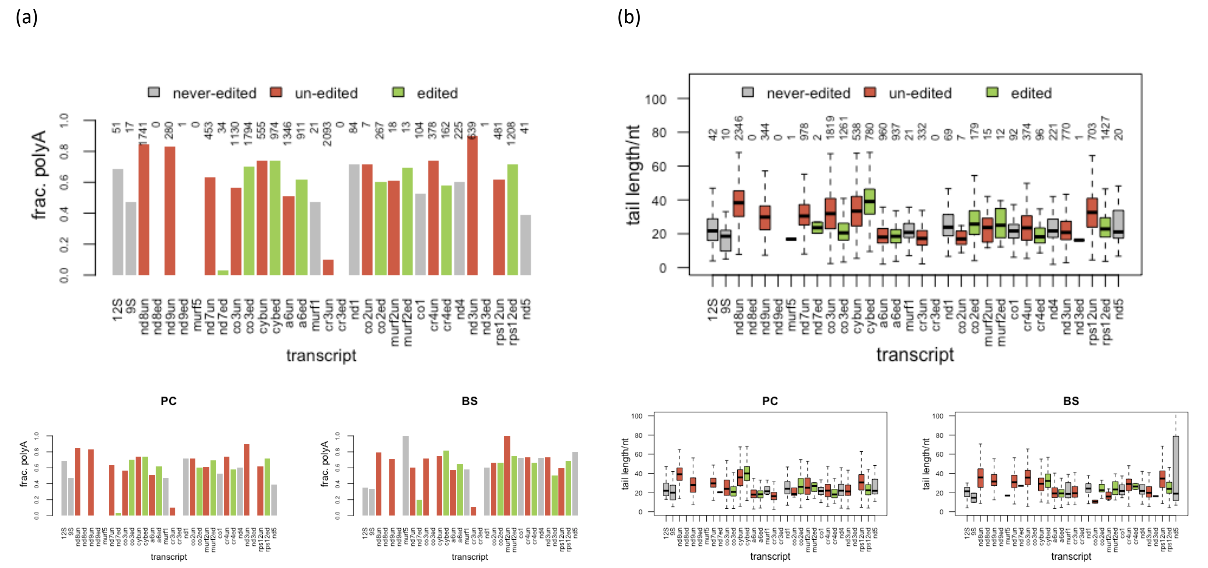

Supplementary Figure S13. Polyadenylation of mitochondrial RNAs. (a) The fraction (frac.) of sequencing reads holding a poly(A)-tail is plotted for all *T. brucei* mitochondrial transcripts. Transcript abbreviations are as in [60]. The total number of reads is indicated by the numbers above each bar. (b) Box-plot of the poly(A)-tail length of all mitochondrial transcripts. Upper panels: Data derived from the combined reads of all DRS libraries (bloodstream-stage (BS) and procyclic-stage (PC). Lower panels: The same data but separated for PC and BS trypanosomes. Grey=never-edited transcripts. Red=un-edited transcripts. Green=edited transcripts.

2. Supplementary Tables

Supplementary Table S1. Read statistics of the DRS sequencing data. Base calling was performed post-sequencing using the high accuracy (HAC) calling model of Guppy [28]. Only reads with a mean per base quality score (*q*-score) >7 were used for further analysis.

	**procyclic-stage *T. brucei***			**bloodstream-stage *T. brucei***		
	**rep1**	**rep2**	**rep3**	**rep1**	**rep2**	**rep3**
**reads total**	2.85E+05	3.37E+05	3.71E+05	6.02E+05	6.66E+05	4.77E+05
**mean qscore >=7**	2.64E+05	3.10E+05	3.43E+05	5.39E+05	5.67E+05	4.43E+05
	92%	92%	92%	89%	85%	93%
**median qscore**						
pass	10.43	9.86	10.32	10.25	9.65	10.46
fail	5.32	5.95	5.80	5.78	5.50	5.62
**median length**						
pass	779	811	786	825	666	750
fail	283	451	378	319	198	244
**max length**						
pass	11784	12690	14262	12971	8697	7912
fail	14139	10395	25252	21789	18425	15827

Supplementary Table S2. Transcript mapping statistics. Reads with *q*-scores >7 were aligned to a composite reference genome (complete rerefence) consisting of the annotated chromosomes of *T. brucei* TREU927 (TryTrypDBv52), the *T. brucei* mitochondrial (maxicircle) genome (NCBI: nucleotide database, accNo M94286.1), and the sequence of yeast ENO2 used as reference calibration sequence (RCS) during library preparation.

	**procyclic-stage *T. brucei***			**bloodstream-stage *T. brucei***		
	**rep1**	**rep2**	**rep3**	**rep1**	**rep2**	**rep3**
input	2.64E+05	3.10E+05	3.43E+05	5.39E+05	5.67E+05	4.43E+05
**# alignments**						
all alignments	4.18E+05	4.93E+05	5.38E+05	8.17E+05	8.60E+05	6.80E+05
unique reads	2.59E+05	3.03E+05	3.35E+05	5.04E+05	5.24E+05	4.16E+05
**RCS**						
unique reads	9901	12020	9803	16319	4802	19147
**maxicircle genome**						
unique reads	3641	3385	4440	2522	1774	3325
**rRNA**						
unique reads	3771	3643	3705	4236	2351	4295
**median error rate**						
complete reference	7.5%	8.5%	7.7%	8.0%	8.9%	7.4%
T. b. chromosomes	7.5%	8.6%	7.8%	8.0%	9.0%	7.5%
RCS	4.9%	5.9%	5.2%	5.2%	5.9%	4.6%
maxicircle	7.1%	7.8%	7.3%	7.7%	8.2%	7.3%

**genome coverage**						
depth > 0	64%	67%	67%	73%	65%	67%
depth > 4	37%	43%	45%	56%	44%	46%
**annotated exons (N=10728)**						
depth > 0	8463	8551	8559	9018	8904	8899
depth > 4	7049	7487	7664	8436	8187	8238

Supplementary Table S3. Identification of full-length DRS sequencing reads. Full-length reads were identified based on the presence of at least 15nt of the spliced leader (SL)-RNA sequence at the 5'-end (5'-SL) in addition to a 3'-poly(A) sequence identified by the poly(A) module of nanopolish. rep=DRS library replicates of procyclic-stage and bloodstream-stage *T. brucei*.

	**procyclic-stage libraries**			**bloodstream-stage libraries**		
	**rep1**	**rep2**	**rep3**	**rep1**	**rep2**	**rep3**
input	2.59E+05	3.03E+05	3.35E+05	5.04E+05	5.24E+05	4.16E+05
5'-SL	1.53E+05	1.73E+05	1.81E+05	1.86E+05	1.43E+05	1.32E+05
	59%	57%	54%	37%	27%	32%
3'-poly(A)	2.24E+05	2.47E+05	2.85E+05	4.29E+05	4.33E+05	3.50E+05
	87%	82%	85%	85%	83%	84%
5'-SL & 3'-poly(A)	1.37E+05	1.45E+05	1.57E+05	1.62E+05	1.22E+05	1.15E+05
	53%	48%	47%	32%	23%	28%

Supplementary Table S4. Performance of lncRNA prediction tools. DRS sequences (supported by minimally 3 reads) were analyzed for their coding potential using CPC2 [36] and LncFinder [37]. Genomic coordinates of sequences predicted as noncoding were compared to the genome annotation file and checked for overlap with the coding sequence (CDS) of annotated mRNAs.

	**total**	**predicted as noncoding**		
		CPC2	LncFinder	overlap
input	10652	2445	1806	1801
annotated CDS	8496	434	99	96
		17.8%	5.5%	5.3%

gene product	annotated	CPC2	LncFinder	overlap
ribosomal protein	254	37	4	4
histone	64	30	1	0
dynein	45	8	0	0
hypothetical/unspecified	2851	230	80	78

Supplementary Table S5. Genomic locations of novel intergenic lncRNAs. Overlapping transcripts are shaded. LncRNAs of similar sequence but localizing to a different genomic locus are shown in column 6. Results from the differential gene expression analysis are indicated column 7. Transcripts showing at least a 4-fold change in transcript level with adjusted *p*-values <0.05 were considered differentially expressed. With the exception of lncRNA nt_6401.1 all are up-regulated in bloodstream-stage trypanosomes. ND: not determined because of a low read counts (<5 reads in either developmental stage or <15 reads total). ID=identification number. nt=novel transcript.

transcript ID	chromosome	start	end	strand	similar to or overapping with	differentially expressed
nt_194.4	Tb927_01_v5.1	8677	9311	-		yes
nt_200.16	Tb927_01_v5.1	59156	59765	-		ND
nt_200.17	Tb927_01_v5.1	59366	59765	-		yes
nt_202.1	Tb927_01_v5.1	63008	64548	-		no
nt_205.1	Tb927_01_v5.1	72570	74748	-		no
nt_1.2	Tb927_01_v5.1	93131	94573	+		ND
nt_212.5	Tb927_01_v5.1	102280	103253	-		no
nt_213.1	Tb927_01_v5.1	103403	103992	-		ND
nt_215.1	Tb927_01_v5.1	127544	128314	-	nt_10.2	ND
nt_10.2	Tb927_01_v5.1	133736	134603	+	nt_215.1	yes
nt_222.2	Tb927_01_v5.1	174593	176674	-		no
nt_17.3	Tb927_01_v5.1	188014	189511	+		yes
nt_249.1	Tb927_01_v5.1	279320	279778	-		ND
nt_255.1	Tb927_01_v5.1	643401	644549	-	nt_256.1	yes
nt_256.1	Tb927_01_v5.1	647417	648547	-	nt_255.1	yes
nt_416.3	Tb927_02_v5.1	4330	6766	-		no
nt_417.1	Tb927_02_v5.1	6890	7248	-		ND
nt_417.2	Tb927_02_v5.1	6890	8350	-		yes
nt_419.1	Tb927_02_v5.1	27741	29774	-		ND
nt_421.1	Tb927_02_v5.1	32945	35103	-		no
nt_423.1	Tb927_02_v5.1	38299	40726	-	nt_430.1	no
nt_430.1	Tb927_02_v5.1	69265	71691	-	nt_423.1	yes
nt_433.3	Tb927_02_v5.1	79896	81446	-		no
nt_445.1	Tb927_02_v5.1	114448	115421	-		yes
nt_447.2	Tb927_02_v5.1	135155	136402	-		yes
nt_451.3	Tb927_02_v5.1	147956	148930	-		yes
nt_468.11	Tb927_02_v5.1	226734	227355	-		ND
nt_300.1	Tb927_02_v5.1	250325	250814	+		no
nt_303.2	Tb927_02_v5.1	257928	258927	+		ND
nt_473.1	Tb927_02_v5.1	274417	275641	-		ND
nt_474.1	Tb927_02_v5.1	301020	301646	-		no
nt_320.1	Tb927_02_v5.1	358649	359954	+		ND
nt_478.1	Tb927_02_v5.1	384788	386406	-		ND
nt_345.1	Tb927_02_v5.1	600480	601455	+	nt_350.1	yes
nt_345.2	Tb927_02_v5.1	600881	601455	+	nt_350.2, nt_350.1, nt_347.2	ND
nt_347.2	Tb927_02_v5.1	604426	604975	+	nt_345.2, nt_345.1, nt_350.2, nt_350.1	yes
nt_350.1	Tb927_02_v5.1	611119	612056	+	nt_345.1	yes
nt_350.2	Tb927_02_v5.1	611510	612056	+	nt_345.2, nt_345.1, nt_347.2	ND
nt_352.1	Tb927_02_v5.1	614653	614930	+	nt_350.1, nt_345.1	ND
nt_596.1	Tb927_02_v5.1	979948	980333	-	nt_375.1	no
nt_599.1	Tb927_02_v5.1	995887	996615	-	nt_373.2	no
nt_373.2	Tb927_02_v5.1	998092	998818	+	nt_599.1	no
nt_373.3	Tb927_02_v5.1	998635	1000193	+		no
nt_373.5	Tb927_02_v5.1	998945	1000193	+		ND
nt_375.1	Tb927_02_v5.1	1014366	1014751	+	nt_596.1	no
nt_401.6	Tb927_02_v5.1	1122445	1123351	+		ND
nt_401.7	Tb927_02_v5.1	1122445	1124737	+		yes
nt_401.8	Tb927_02_v5.1	1123050	1124737	+		ND
nt_401.9	Tb927_02_v5.1	1123464	1124737	+		ND
nt_411.1	Tb927_02_v5.1	1164676	1166191	+		ND

nt_413.2	Tb927_02_v5.1	1168338	1168836	+		ND
nt_413.3	Tb927_02_v5.1	1168338	1169283	+		ND
nt_841.3	Tb927_03_v5.1	384581	385789	-		ND
nt_844.1	Tb927_03_v5.1	391980	393761	-		yes
nt_990.4	Tb927_03_v5.1	1160150	1160561	-		no
nt_1160.1	Tb927_04_v5.1	199	700	-		ND
nt_1161.1	Tb927_04_v5.1	815	1287	-		no
nt_1162.2	Tb927_04_v5.1	7423	8867	-		no
nt_1162.3	Tb927_04_v5.1	7888	8867	-		ND
nt_1107.1	Tb927_04_v5.1	692681	693120	+		no
nt_1399.1	Tb927_04_v5.1	1172047	1172332	-		ND
nt_1158.1	Tb927_04_v5.1	1472777	1474585	+		ND
nt_1158.4	Tb927_04_v5.1	1474722	1476645	+		ND
nt_1742.1	Tb927_05_v5.1	13140	14681	-		yes
nt_1742.3	Tb927_05_v5.1	13807	14681	-		ND
nt_1743.1	Tb927_05_v5.1	15984	19513	-		yes
nt_1744.1	Tb927_05_v5.1	19648	20803	-		no
nt_1744.6	Tb927_05_v5.1	20808	22315	-		no
nt_1487.1	Tb927_05_v5.1	27791	28386	+		yes
nt_1745.2	Tb927_05_v5.1	66202	68362	-	nt_1493.4, nt_1493.5	no
nt_1750.1	Tb927_05_v5.1	83729	84843	-	nt_1762.2	ND
nt_1750.2	Tb927_05_v5.1	83729	86415	-		no
nt_1489.2	Tb927_05_v5.1	100971	101950	+		ND
nt_1492.1	Tb927_05_v5.1	108791	109331	+		yes
nt_1493.1	Tb927_05_v5.1	113480	114387	+		ND
nt_1493.2	Tb927_05_v5.1	113480	115188	+		ND
nt_1493.3	Tb927_05_v5.1	114523	115188	+		no
nt_1493.4	Tb927_05_v5.1	114523	117496	+		ND
nt_1493.5	Tb927_05_v5.1	115337	117496	+	nt_1745.2	no
nt_1493.6	Tb927_05_v5.1	116306	117496	+	nt_1745.2	ND
nt_1759.1	Tb927_05_v5.1	134729	135378	-		yes
nt_1762.2	Tb927_05_v5.1	146145	148907	-		no
nt_1763.1	Tb927_05_v5.1	149009	149832	-		no
nt_1768.1	Tb927_05_v5.1	169875	170775	-		ND
nt_1768.2	Tb927_05_v5.1	169875	171185	-		ND
nt_1768.4	Tb927_05_v5.1	169875	172240	-		ND
nt_1768.7	Tb927_05_v5.1	170931	172240	-		ND
nt_1768.8	Tb927_05_v5.1	171302	172240	-		ND
nt_1500.1	Tb927_05_v5.1	176139	176983	+		ND
nt_1769.1	Tb927_05_v5.1	184812	185584	-		ND
nt_1504.1	Tb927_05_v5.1	249055	249330	+		ND
nt_1527.1	Tb927_05_v5.1	353506	353925	+		no
nt_1667.1	Tb927_05_v5.1	929096	929834	+		ND
nt_1725.1	Tb927_05_v5.1	1395730	1396781	+		no
nt_1728.1	Tb927_05_v5.1	1409234	1410517	+		no
nt_1729.4	Tb927_05_v5.1	1416865	1417172	+		no
nt_1737.1	Tb927_05_v5.1	1435122	1436168	+		ND
nt_1738.1	Tb927_05_v5.1	1438285	1439317	+		ND
nt_1922.3	Tb927_06_v5.1	22976	23454	+		ND
nt_1922.4	Tb927_06_v5.1	22976	24015	+		no
nt_2127.1	Tb927_06_v5.1	32116	32745	-		no
nt_2128.1	Tb927_06_v5.1	32860	33654	-		no
nt_2129.1	Tb927_06_v5.1	33779	34307	-	nt_2132.4, nt_1935.1	ND
nt_1923.3	Tb927_06_v5.1	38192	39606	+		no
nt_1923.5	Tb927_06_v5.1	39808	41546	+	nt_2143.1	no
nt_2132.4	Tb927_06_v5.1	77739	78283	-	nt_1935.1, nt_2129.1	no
nt_1928.1	Tb927_06_v5.1	90325	91026	+	nt_2136.1, nt_2136.3, nt_2140.1, nt_2140.2, nt_1940.2	no
nt_2136.1	Tb927_06_v5.1	117583	120484	-		no
nt_2136.3	Tb927_06_v5.1	119616	120484	-	nt_2140.2, nt_2140.1	ND
nt_2138.1	Tb927_06_v5.1	125083	125343	-	nt_2142.1	ND
nt_2140.1	Tb927_06_v5.1	130027	131874	-	nt_2136.1	ND

nt_2140.2	Tb927_06_v5.1	130996	131874	-	nt_2136.3, nt_2136.1, nt_1940.2	ND
nt_2140.3	Tb927_06_v5.1	131458	131874	-	nt_2136.3, nt_2136.1, nt_1928.1	ND
nt_2142.1	Tb927_06_v5.1	136469	136723	-	nt_2138.1	ND
nt_2143.1	Tb927_06_v5.1	141355	143137	-	nt_1923.5	no
nt_1935.1	Tb927_06_v5.1	148639	149183	+	nt_2132.4	no
nt_1937.1	Tb927_06_v5.1	150201	150812	+		no
nt_1940.2	Tb927_06_v5.1	160294	161157	+	nt_2140.2, nt_2140.1, nt_2136.3, nt_2136.1	ND
nt_1965.1	Tb927_06_v5.1	339689	340081	+		ND
nt_2178.1	Tb927_06_v5.1	363244	363619	-		ND
nt_1983.1	Tb927_06_v5.1	728981	729771	+		ND
nt_2374.1	Tb927_07_v5.1	32685	34046	+		ND
nt_2707.1	Tb927_07_v5.1	479102	479709	-	nt_2710.1, nt_2713.1	ND
nt_2710.1	Tb927_07_v5.1	484661	485296	-	nt_2707.1, nt_2713.1	ND
nt_2713.1	Tb927_07_v5.1	501461	502096	-	nt_2710.1, nt_2707.1	ND
nt_2726.4	Tb927_07_v5.1	543327	543837	-		ND
nt_2496.1	Tb927_07_v5.1	834988	836167	+		ND
nt_2856.1	Tb927_07_v5.1	1238481	1239149	-		ND
nt_2875.1	Tb927_07_v5.1	1285552	1286087	-		no
nt_2881.1	Tb927_07_v5.1	1308199	1309532	-		no
nt_2615.1	Tb927_07_v5.1	1601963	1602373	+		no
nt_2633.1	Tb927_07_v5.1	1790335	1790900	+		yes
nt_2926.1	Tb927_07_v5.1	1839125	1839473	-		ND
nt_2983.1	Tb927_07_v5.1	2096938	2097338	-		ND
nt_2983.3	Tb927_07_v5.1	2096947	2097338	-		ND
nt_3001.1	Tb927_08_v5.1	49746	50574	+		ND
nt_3446.1	Tb927_08_v5.1	296504	297024	-		no
nt_3073.1	Tb927_08_v5.1	791866	792596	+		ND
nt_3089.1	Tb927_08_v5.1	856518	857230	+		ND
nt_3144.4	Tb927_08_v5.1	1045706	1046768	+		ND
nt_3511.1	Tb927_08_v5.1	1056525	1057061	-		ND
nt_3541.1	Tb927_08_v5.1	1155854	1157057	-		no
nt_3260.1	Tb927_08_v5.1	1998335	1999109	+		no
nt_3316.1	Tb927_08_v5.1	2201558	2201957	+	nt_3320.1, nt_3318.2	ND
nt_3318.2	Tb927_08_v5.1	2204283	2204682	+	nt_3320.1, nt_3316.1	ND
nt_3320.1	Tb927_08_v5.1	2207008	2207407	+	nt_3318.2, nt_3316.1	ND
nt_3670.1	Tb927_08_v5.1	2236556	2237618	-		ND
nt_3396.1	Tb927_08_v5.1	2459446	2459948	+		ND
nt_4004.1	Tb927_09_v5.1	158853	159499	-		yes
nt_4020.1	Tb927_09_v5.1	351703	352172	-		ND
nt_4082.1	Tb927_09_v5.1	613931	614541	-	nt_4099.1, nt_4089.1, nt_4091.1	ND
nt_4089.1	Tb927_09_v5.1	626134	626762	-	nt_4091.1, nt_4099.1, nt_4082.1	no
nt_4091.1	Tb927_09_v5.1	629231	629858	-	nt_4089.1, nt_4099.1, nt_4082.1	no
nt_4099.1	Tb927_09_v5.1	648586	649212	-	nt_4082.1, nt_4091.1, nt_4089.1	ND
nt_4118.1	Tb927_09_v5.1	1189203	1189814	-	nt_4124.3	ND
nt_4120.1	Tb927_09_v5.1	1192799	1195231	-		yes
nt_4120.2	Tb927_09_v5.1	1194028	1195231	-		ND
nt_4124.3	Tb927_09_v5.1	1211124	1211735	-	nt_4118.1	ND
nt_4146.1	Tb927_09_v5.1	1303364	1304868	-		ND
nt_4220.1	Tb927_09_v5.1	1525948	1526948	-		no
nt_4243.7	Tb927_09_v5.1	1611151	1611571	-		ND
nt_3837.1	Tb927_09_v5.1	1824285	1824705	+		ND
nt_4296.1	Tb927_09_v5.1	2087164	2088287	-		no
nt_3936.1	Tb927_09_v5.1	2286546	2287343	+		ND
nt_3972.4	Tb927_09_v5.1	2440372	2440616	+	nt_3975.1	no
nt_3975.1	Tb927_09_v5.1	2443945	2444189	+	nt_3972.4	no
nt_3976.5	Tb927_09_v5.1	2445730	2446145	+		no
nt_3977.3	Tb927_09_v5.1	2448087	2448768	+		no
nt_4363.1	Tb927_09_v5.1	2512687	2513703	-		ND

nt_4363.2	Tb927_09_v5.1	2512687	2515347	-		ND
nt_3987.6	Tb927_09_v5.1	2523052	2524193	+		yes
nt_4377.1	Tb927_09_v5.1	2668064	2669227	-		ND
nt_4381.1	Tb927_09_v5.1	2678157	2678924	-		ND
nt_3994.1	Tb927_09_v5.1	2690171	2690831	+		ND
nt_3995.1	Tb927_09_v5.1	2692919	2694243	+		ND
nt_3996.1	Tb927_09_v5.1	2696153	2697089	+		ND
nt_4397.1	Tb927_09_v5.1	3405122	3406513	-		ND
nt_5045.1	Tb927_10_v5.1	21824	23765	-		yes
nt_5049.1	Tb927_10_v5.1	37614	38106	-		ND
nt_4426.1	Tb927_10_v5.1	140102	140474	+		ND
nt_4456.1	Tb927_10_v5.1	257007	257389	+		no
nt_5077.1	Tb927_10_v5.1	365551	366118	-		no
nt_5083.1	Tb927_10_v5.1	391955	392513	-		ND
nt_5089.2	Tb927_10_v5.1	425194	425582	-		ND
nt_4462.1	Tb927_10_v5.1	452242	453144	+		no
nt_5101.1	Tb927_10_v5.1	470525	471426	-		no
nt_4504.1	Tb927_10_v5.1	580560	581061	+		ND
nt_4515.1	Tb927_10_v5.1	612769	613390	+		ND
nt_4554.1	Tb927_10_v5.1	745710	746110	+		no
nt_4561.6	Tb927_10_v5.1	777032	778057	+		no
nt_4580.1	Tb927_10_v5.1	837019	837242	+		ND
nt_4638.3	Tb927_10_v5.1	1289793	1290560	+		no
nt_4641.4	Tb927_10_v5.1	1302543	1303880	+		no
nt_4642.1	Tb927_10_v5.1	1304003	1304550	+		ND
nt_5222.1	Tb927_10_v5.1	1493180	1493729	-		ND
nt_5229.4	Tb927_10_v5.1	1527472	1529007	-		no
nt_4701.3	Tb927_10_v5.1	1684401	1685118	+		ND
nt_4721.4	Tb927_10_v5.1	1731709	1732213	+		no
nt_4739.1	Tb927_10_v5.1	1787996	1788361	+		ND
nt_5285.4	Tb927_10_v5.1	1885770	1886833	-		ND
nt_5285.5	Tb927_10_v5.1	1885770	1887510	-		ND
nt_5302.1	Tb927_10_v5.1	1944645	1945114	-		no
nt_5319.8	Tb927_10_v5.1	1999712	2000212	-		no
nt_4823.1	Tb927_10_v5.1	2334878	2336931	+		yes
nt_4829.2	Tb927_10_v5.1	2349525	2350028	+		yes
nt_5373.4	Tb927_10_v5.1	2639414	2641305	-		no
nt_5373.5	Tb927_10_v5.1	2639674	2641305	-		ND
nt_5373.7	Tb927_10_v5.1	2640254	2641305	-		ND
nt_5374.1	Tb927_10_v5.1	2641396	2642378	-		ND
nt_5374.2	Tb927_10_v5.1	2641396	2642574	-		no
nt_5387.1	Tb927_10_v5.1	2688192	2688712	-		no
nt_4896.11	Tb927_10_v5.1	2828919	2829983	+		ND
nt_5425.1	Tb927_10_v5.1	3027811	3028219	-		ND
nt_5425.2	Tb927_10_v5.1	3027811	3028626	-		ND
nt_5425.3	Tb927_10_v5.1	3027866	3028626	-		no
nt_5504.1	Tb927_10_v5.1	3302765	3303306	-		no
nt_5600.9	Tb927_10_v5.1	3616215	3616803	-		no
nt_5601.1	Tb927_10_v5.1	3616907	3617888	-		ND
nt_5601.2	Tb927_10_v5.1	3616907	3619154	-		no
nt_5601.4	Tb927_10_v5.1	3618107	3619154	-		ND
nt_4981.3	Tb927_10_v5.1	3724065	3724420	+		ND
nt_5003.1	Tb927_10_v5.1	3813154	3813738	+		no
nt_5013.1	Tb927_10_v5.1	3865755	3866225	+		no
nt_5027.3	Tb927_10_v5.1	3924719	3925126	+		ND
nt_6411.1	Tb927_11_v5.1	29046	30954	-	nt_5697.2	no
nt_5691.2	Tb927_11_v5.1	421251	422132	+		no
nt_5697.1	Tb927_11_v5.1	444818	445778	+	nt_6411.1	ND
nt_5697.2	Tb927_11_v5.1	444818	446739	+	nt_6411.1	ND
nt_6484.6	Tb927_11_v5.1	551680	552075	-		ND
nt_6484.7	Tb927_11_v5.1	551680	552602	-		no
nt_6484.8	Tb927_11_v5.1	552195	552602	-		ND

nt_6556.1	Tb927_11_v5.1	917913	918913	-		ND
nt_5872.4	Tb927_11_v5.1	1404891	1405479	+		no
nt_6609.4	Tb927_11_v5.1	1751495	1751803	-		ND
nt_6689.1	Tb927_11_v5.1	2023993	2025461	-		yes
nt_5965.4	Tb927_11_v5.1	2046931	2047701	+		no
nt_5989.1	Tb927_11_v5.1	2110152	2110889	+		ND
nt_6236.1	Tb927_11_v5.1	3149663	3150319	+		no
nt_6773.2	Tb927_11_v5.1	3206614	3208824	-		no
nt_6773.12	Tb927_11_v5.1	3209915	3210568	-		no
nt_6256.1	Tb927_11_v5.1	3214193	3215719	+		no
nt_6258.1	Tb927_11_v5.1	3216967	3217711	+		ND
nt_6258.2	Tb927_11_v5.1	3216967	3218598	+		ND
nt_6807.1	Tb927_11_v5.1	3515911	3517185	-		no
nt_6809.1	Tb927_11_v5.1	3521361	3522957	-		no
nt_6956.1	Tb927_11_v5.1	4193182	4193957	-		no
nt_6956.2	Tb927_11_v5.1	4193430	4193957	-	nt_6960.1	ND
nt_6960.1	Tb927_11_v5.1	4199973	4200510	-	nt_6956.1, nt_6956.2	ND
nt_6401.1	Tb927_11_v5.1	4839999	4841142	+		yes *)
nt_5625.2	Tb927_11_RH_fork_v5.1	136516	137375	-		ND
nt_5630.1	Tb927_11_RH_fork_v5.1	237731	238549	-		ND
nt_5631.1	Tb927_11_RH_fork_v5.1	617151	618126	-		yes

*) up-regulated in procyclic-stage trypanosomes

Supplementary Table S6. Differential gene expression. Genes up-regulated in bloodstream-stage trypanosomes.

**gene-ID**	**fold change (BS/PC)**	**adjusted *p*-value**	**gene product**
Tb927.9.16490	143.20	7.21E-23	variant surface glycoprotein (VSG), putative
Tb927.1.20	132.70	2.47E-21	expression site-associated gene 3 (ESAG3), pseudogene
Tb927.1.5100	97.18	2.82E-16	expression site-associated gene 2 (ESAG2) protein, putative
Tb927.1.4910	95.78	1.59E-16	expression site-associated gene 1 (ESAG1) protein, putative
Tb927.5.150	79.35	3.12E-13	hypothetical protein, conserved
Tb927.2.3320	75.78	8.34E-13	65 kDa invariant surface glycoprotein
Tb11.01.6240	71.05	9.28E-12	expression site-associated gene 2 (ESAG2) protein, putative
nt_5631.1	70.64	1.43E-11	lncRNA, putative
Tb11.01.6250	69.34	3.92E-11	expression site-associated gene 11 (ESAG11) protein, putative
Tb927.7.170	69.15	6.91E-11	expression site-associated gene 9 (ESAG9) protein, putative
Tb927.1.5110	68.85	1.67E-11	expression site-associated gene 11 (ESAG11) protein, putative
Tb927.9.7380	68.60	2.54E-11	variant surface glycoprotein (VSG)-related, putative
Tb927.5.1390	66.47	8.13E-11	64 kDa invariant surface glycoprotein
Tb927.5.120	66.34	1.57E-10	expression site-associated gene 9 (ESAG9) protein, putative
Tb927.2.6180	66.20	2.50E-10	iron/ascorbate oxidoreductase family protein, putative
Tb927.1.4870	65.73	1.33E-10	expression site-associated gene 1 (ESAG1) protein, putative
Tb927.9.7320	64.18	6.15E-10	expression site-associated gene 11 (ESAG11) protein, putative
Tb927.1.4900	64.07	1.28E-18	expression site-associated gene 11 (ESAG11) protein, putative
Tb927.9.16880	63.67	4.01E-10	expression site-associated gene 3 (ESAG3, pseudogene), putative
Tb927.9.16500	63.22	3.16E-09	variant surface glycoprotein (VSG, atypical), putative
Tb927.11.4100	63.06	5.74E-10	variant surface glycoprotein (VSG), putative
nt_194.4	62.33	1.04E-09	lncRNA, putative
Tb927.3.570	61.02	2.87E-09	expression site-associated gene 2 (ESAG2) protein, putative
KS17gene_223a	61.01	1.37E-09	lncRNA, putative
Tb927.3.1520	60.61	1.34E-08	variant surface glycoprotein (VSG)-related, putative
Tb927.2.3310	59.88	2.43E-09	65 kDa invariant surface glycoprotein
Tb927.3.560	59.36	5.15E-08	expression site-associated gene 11 (ESAG11) protein, putative
Tb927.5.1400	58.96	1.33E-08	hypothetical protein
Tb927.5.4900	58.55	4.22E-07	variant surface glycoprotein, frameshift
Tb927.7.160	58.27	9.32E-09	expression site-associated gene 2 (ESAG2), degenerate
Tb927.11.14610	57.96	1.49E-08	procyclin-associated gene 4 (PAG4) protein, putative
Tb927.1.5120	57.53	1.11E-08	expression site-associated gene 1 (ESAG1) protein, putative
Tb927.1.5240	56.62	1.96E-08	expression site-associated gene 1 (ESAG1) protein, putative
nt_5045.1	56.58	1.64E-08	lncRNA, putative
Tb927.2.6320	56.50	2.66E-08	adenosine transporter 2, putative
Tb927.2.3295	55.98	5.53E-08	unspecified product
Tb927.8.7330	55.59	3.91E-08	hypothetical protein
Tb927.11.14620	55.17	9.38E-17	expression site-associated gene 2 (ESAG2) protein, putative
KS17gene_225a	54.70	4.96E-08	lncRNA, putative
Tb927.3.1470	53.95	7.93E-08	variant surface glycoprotein (VSG)-related, putative
nt_4829.2	53.75	9.41E-08	lncRNA, putative
Tb927.9.7340	52.73	2.04E-07	expression site-associated gene 9 (ESAG9) protein, putative
Tb927.5.1410	52.09	9.16E-07	64 kDa invariant surface glycoprotein
Tb927.3.5790	50.15	6.84E-07	expression site-associated gene 9 (ESAG9) (pseudogene)
KS17gene_6206a	50.10	1.90E-06	lncRNA, putative
Tb927.3.5830	50.07	7.94E-07	expression site-associated gene 1 (ESAG1) protein, putative
Tb927.9.7410	49.98	1.34E-06	expression site-associated gene 2 (ESAG2) protein, putative
Tb927.11.14600	49.96	1.27E-06	procyclin-associated gene 2-like protein, putative
Tb927.5.5450	49.92	7.96E-07	Variant Surface Glycoprotein, putative
nt_1742.1	49.75	8.78E-07	lncRNA, putative
KS17gene_2863a	49.46	1.00E-06	lncRNA, putative
Tb927.3.1500	49.14	1.79E-06	variant surface glycoprotein (VSG)-related, putative
Tb927.11.18660	48.96	1.19E-06	expression site-associated gene 9 (ESAG9), putative (pseudogene)
Tb927.9.730	48.87	1.54E-06	hypothetical protein, conserved
nt_347.2	48.65	2.07E-06	lncRNA, putative
Tb927.2.3315	48.63	6.93E-06	unspecified product
Tb927.10.1780	48.23	3.59E-06	hypothetical protein

Tb927.3.5820	48.17	2.41E-06	expression site-associated gene 11 (ESAG11), degenerate
Tb927.10.5680	47.98	2.00E-06	procyclin-associated gene 1 (PAG1) protein, putative
Tb927.3.2590	47.92	2.22E-06	hypothetical protein
Tb927.5.1420	46.99	3.98E-06	hypothetical protein
KS17gene_3556a	46.91	4.01E-06	lncRNA, putative
KS17gene_3552a	46.64	3.81E-06	lncRNA, putative
Tb09_snoRNA_0076	46.30	8.06E-06	H/ACA snoRNA, TB9Cs2H1
Tb927.9.2855	45.64	7.25E-06	Domain of unknown function (DUF5075), putative
nt_844.1	45.51	9.08E-06	lncRNA, putative
Tb927.2.6230	45.25	7.82E-06	iron/ascorbate oxidoreductase family protein, putative
Tb927.8.1665	45.16	9.22E-06	hypothetical protein
Tb927.1.4890	45.07	1.14E-13	expression site-associated gene 2 (ESAG2) protein, putative
Tb927.6.1340	44.80	1.25E-05	cyclophilin-type peptidyl-prolyl cis-trans isomerase, putative
Tb927.2.200	44.28	4.05E-18	expression site-associated gene 3 (ESAG3), degenerate
Tb927.3.5810	44.22	1.10E-05	expression site-associated gene 2 (ESAG2) (pseudogene)
Tb927.3.520	44.15	1.59E-05	expression site-associated gene 1 (ESAG1) protein, putative
KS17gene_943a	42.59	2.39E-05	lncRNA, putative
Tb927.1.5170	41.74	4.45E-05	variant surface glycoprotein (VSG)-related, putative
Tb927.11.12120	41.53	4.71E-05	RNA-binding protein, putative
Tb927.5.110	41.47	3.68E-05	variant surface glycoprotein (VSG)-related, putative
KS17gene_6881a	41.26	7.86E-05	lncRNA, putative
Tb927.6.2520	41.03	5.23E-05	hypothetical protein, conserved
Tb927.7.3260	40.63	9.12E-17	expression site-associated gene 7 (ESAG7) protein, putative
Tb927.5.130	40.20	1.15E-04	variant surface glycoprotein (VSG)-related, putative
Tb927.3.5800	39.77	1.29E-04	expression site-associated gene 1 (ESAG1), degenerate
Tb927.2.3270	39.38	1.11E-17	65 kDa invariant surface glycoprotein
Tb927.5.1430	38.82	1.30E-04	64 kDa invariant surface glycoprotein
nt_4004.1	38.77	1.06E-04	lncRNA, putative
nt_350.1	37.98	2.55E-04	lncRNA, putative
Tb927.2.3280	37.74	3.01E-04	65 kDa invariant surface glycoprotein
nt_401.7	37.29	2.07E-04	lncRNA, putative
Tb927.11.18650	36.22	4.15E-04	expression site-associated gene 1 (ESAG1) protein, putative
Tb927.2.6220	35.81	4.12E-04	adenosine transporter 2, putative
Tb927.9.7350	35.36	4.30E-04	expression site-associated gene 1 (ESAG1), pseudogene
Tb927.6.1390	35.21	4.61E-04	Trypanosomal VSG domain containing protein, putative
KS17gene_4587a	34.85	5.86E-04	lncRNA, putative
Tb927.9.7290	33.63	7.73E-04	variant surface glycoprotein (VSG)-related, putative
nt_2633.1	33.55	8.45E-04	lncRNA, putative
Tb927.2.3290	33.51	2.27E-15	65 kDa invariant surface glycoprotein
KS17gene_202a	32.37	1.16E-03	lncRNA, putative
KS17gene_4541a	32.37	1.11E-03	lncRNA, putative
Tb927.7.7510	32.35	1.63E-24	hypothetical protein
Tb927.3.550	31.71	1.47E-03	expression site-associated gene 1 (ESAG1), degenerate
KS17gene_7015a	31.59	9.15E-13	lncRNA, putative
Tb927.2.1380	31.33	1.57E-09	leucine-rich repeat protein (LRRP), putative
Tb927.6.340	31.15	1.71E-03	receptor-type adenylate cyclase GRESAG 4, pseudogene, putative
Tb927.2.2029	30.96	1.93E-09	expression site-associated gene 3 (ESAG3), degenerate
nt_345.1	30.42	2.11E-03	lncRNA, putative
Tb927.7.3250	30.34	9.81E-15	expression site-associated gene 6 (ESAG6) protein, putative
Tb927.4.230	30.21	4.18E-09	DNA-directed RNA polymerase III subunit, pseudogene, putative
Tb927.5.309b	30.14	2.22E-12	invariant surface glycoprotein, putative
KS17gene_215a	28.57	9.06E-12	lncRNA, putative
Tb927.10.1480	28.39	2.61E-08	hypothetical protein
Tb927.10.9450	27.25	4.98E-13	invariant surface glycoprotein, putative
Tb927.11.17890	26.97	6.95E-13	expression site-associated gene 1 (ESAG1) protein, putative
Tb927.10.5710	26.14	1.88E-12	hypothetical protein, conserved
Tb927.5.400	26.09	1.64E-10	75 kDa invariant surface glycoprotein, putative
Tb927.4.200	26.00	1.24E-07	retrotransposon hot spot protein 1 (RHS1), putative
Tb927.6.1040	25.35	7.92E-15	cysteine peptidase, Clan CA, family C1, Cathepsin L-like
Tb927.6.5180	25.09	3.68E-15	retrotransposon hot spot protein 4 (RHS4), interrupted
Tb927.1.4860	24.90	4.49E-07	expression site-associated gene 11 (ESAG11), pseudogene
Tb927.4.1230	24.38	4.77E-17	hypothetical protein
nt_4823.1	24.32	5.15E-07	lncRNA, putative

Tb927.10.12800	23.88	1.09E-08	Zinc finger CCCH domain-containing protein 38
Tb927.9.16050	23.29	1.39E-06	leucine-rich repeat protein (pseudogene), putative
Tb927.7.180	22.61	1.14E-05	Trypanosomal VSG domain containing protein, putative
KS17gene_5352a	21.57	6.56E-11	lncRNA, putative
Tb927.5.160	21.44	5.25E-06	retrotransposon hot spot protein (RHS, pseudogene), putative
KS17gene_7014a	21.22	1.08E-05	lncRNA, putative
KS17gene_265a	21.12	6.56E-11	lncRNA, putative
Tb927.8.6760	20.76	9.23E-15	translationally-controlled tumor protein homolog, putative
Tb927.10.5690	20.67	2.38E-08	procyclin-associated gene 2 (PAG2) protein, putative
Tb927.6.1000	20.05	6.01E-16	cysteine peptidase, Clan CA, family C1, Cathepsin L-like
Tb927.11.2400	19.73	1.90E-07	Flabarin-like protein
Tb927.2.3340	19.45	9.43E-08	hypothetical protein
nt_3987.6	18.58	1.20E-07	lncRNA, putative
Tb927.11.4120	18.50	5.35E-05	leucine-rich repeat protein (LRRP), putative
Tb927.2.980	18.40	3.51E-07	retrotransposon hot spot protein 5 (RHS5), degenerate
Tb927.10.9510	18.19	8.66E-05	hypothetical protein
KS17gene_212a	18.10	7.64E-05	lncRNA, putative
Tb927.1.5060	17.86	1.53E-08	variant surface glycoprotein (VSG)-related, putative
KS17gene_4915a	17.77	1.27E-04	lncRNA, putative
Tb927.1.5200	17.68	9.83E-07	expression site-associated gene 1 (ESAG1) protein, putative
Tb927.9.15680	17.49	1.03E-04	expression site-associated gene 6 (ESAG6)
Tb927.10.10360	17.15	8.24E-07	Microtubule-associated repetitive protein
nt_200.17	16.85	9.44E-07	lncRNA, putative
Tb927.6.140	16.68	4.00E-12	retrotransposon hot spot protein 5 (RHS5), putative
Tb927.3.4070	16.67	5.16E-18	Pyruvate transporter, putative
nt_1487.1	16.39	3.40E-08	lncRNA, putative
Tb927.1.2040	15.84	3.48E-04	expression site-associated gene 2 (ESAG2) protein, putative
Tb927.6.330	15.78	8.88E-06	receptor-type adenylate cyclase GRESAG 4, putative
Tb927.11.17880	15.66	4.19E-04	expression site-associated gene 9 (ESAG9), degenerate
Tb927.9.15940	15.66	5.02E-04	expression site-associated gene 3 (ESAG3) protein, putative
Tb927.7.370	15.62	4.41E-13	hypothetical protein, conserved
KS17gene_2525a	15.53	4.96E-04	lncRNA, putative
Tb927.4.5260	15.27	1.65E-05	UDP-Gal or UDP-GlcNAc-dependent glycosyltransferase, putative
Tb927.10.9465	15.23	1.36E-05	hypothetical protein
Tb927.3.5690	15.13	8.84E-08	hypothetical protein, conserved
Tb927.6.990	14.97	1.49E-14	cysteine peptidase, Clan CA, family C1, Cathepsin L-like
Tb927.1.700	14.89	1.94E-16	phosphoglycerate kinase
Tb927.4.4860	14.75	9.63E-16	amino acid transporter 8, putative
KS17gene_4604a	14.17	5.84E-12	lncRNA, putative
Tb927.6.1050	14.07	4.51E-14	cysteine peptidase, Clan CA, family C1, Cathepsin L-like
Tb927.5.293b	14.05	5.61E-12	hypothetical protein
Tb927.2.960	13.93	9.09E-09	hypothetical protein
Tb927.7.6060	13.89	1.16E-03	receptor-type adenylate cyclase GRESAG 4, putative
nt_4120.1	13.87	1.45E-03	lncRNA, putative
Tb927.6.210	13.85	1.53E-03	leucine-rich repeat protein (LRRP, pseudogene), putative
KS17gene_4363a	13.65	1.36E-03	lncRNA, putative
KS17gene_6546a	13.57	1.52E-03	lncRNA, putative
nt_447.2	13.04	2.34E-03	lncRNA, putative
KS17gene_4610a	12.97	1.40E-04	lncRNA, putative
Tb927.11.11975	12.93	5.24E-15	cytoskeleton-associated protein, putative
Tb927.1.430	12.92	1.28E-04	retrotransposon hot spot protein (RHS, pseudogene), putative
Tb927.3.600	12.85	5.15E-11	hypothetical protein
Tb927.5.4600	12.83	8.06E-09	expression site-associated gene 3 (ESAG3) protein, putative
Tb927.2.490	12.77	4.40E-10	DNA-directed RNA polymerase, pseudogene, putative
Tb8.NT.18	12.39	3.46E-03	lncRNA, putative
Tb927.9.10650	12.02	9.09E-09	hypothetical protein
Tb927.7.5790	11.92	2.16E-10	protein disulfide isomerase, putative
Tb927.11.13630	11.90	1.17E-05	hypothetical protein, conserved
Tb927.11.8490	11.77	2.15E-04	DNA polymerase kappa, putative
Tb927.2.1330	11.59	2.16E-14	retrotransposon hot spot protein 6 (RHS6), degenerate
Tb927.5.340	11.54	2.46E-06	expression site-associated gene 5 (ESAG5) protein, putative
Tb927.4.4780	11.53	2.82E-09	hypothetical protein
Tb927.8.3720	11.37	8.72E-10	SUMO-interacting motif-containing protein

Tb927.9.7270	11.35	1.57E-06	hypothetical protein
KS17gene_7564a	11.28	5.81E-03	lncRNA, putative
Tb927.1.1740	11.23	2.99E-04	Microtubule-associated protein futsch, putative
nt_1492.1	11.12	3.67E-04	lncRNA, putative
Tb927.7.7520	10.95	1.36E-15	receptor-type adenylate cyclase GRESAG 4, putative
KS17gene_6494a	10.91	6.62E-14	lncRNA, putative
Tb927.6.350	10.68	5.89E-04	hypothetical protein, conserved
Tb927.9.660	10.59	8.70E-03	expression site-associated gene 1 (ESAG1) protein
Tb927.11.12130	10.55	8.51E-03	hypothetical protein
Tb927.2.1110	10.48	4.80E-08	DNA-directed RNA polymerase III subunit 2, pseudogene, putative
Tb927.8.5465	10.47	1.17E-10	flagellar calcium-binding 24 kDa protein
Tb927.2.2020	10.35	3.23E-06	expression site-associated gene 3 (ESAG3) protein, putative
Tb927.4.4870	10.32	2.54E-14	amino acid transporter, putative
Tb927.9.740	10.23	5.55E-06	hypothetical protein, conserved
Tb927.4.4450	10.13	9.29E-09	adenylyl cyclase
Tb927.7.6500	10.12	1.68E-04	variant surface glycoprotein (VSG), putative
Tb927.8.4110	10.01	8.47E-11	Flagellum adhesion protein 3, putative
Tb927.8.1945	9.89	2.09E-10	hypothetical protein
nt_10.2	9.83	1.24E-02	lncRNA, putative
nt_1743.1	9.82	2.38E-07	lncRNA, putative
Tb927.2.420	9.82	2.03E-13	DNA-directed RNA polymerase, pseudogene, putative
Tb927.11.7550	9.79	2.81E-11	hypothetical protein, conserved
Tb927.10.14890	9.73	2.48E-14	C-terminal motor kinesin, putative
Tb927.5.4630	9.63	1.68E-04	expression site-associated gene 1 (ESAG1) protein, putative
Tb927.5.4570	9.62	1.40E-06	Flagellum adhesion protein 3
Tb927.2.540	9.62	1.06E-13	DNA-directed RNA polymerase, pseudogene, putative
Tb927.4.4420	9.61	1.10E-03	adenylyl cyclase, pseudogene, putative
Tb07.30D13.110	9.47	2.32E-07	hypothetical protein, conserved (pseudogene)
Tb927.5.4010	9.45	1.10E-09	Enriched in surface-labeled proteome protein 4
Tb927.7.390	9.32	2.30E-05	hypothetical protein, conserved
Tb2.NT.8	9.27	6.77E-06	lncRNA, putative
Tb927.5.310	9.26	7.17E-11	invariant surface glycoprotein, putative
KS17gene_6111a	9.22	2.60E-03	lncRNA, putative
Tb927.7.2970	9.17	7.05E-07	ATP-dependent DEAD/H RNA helicase, putative
Tb927.2.6000	9.10	9.78E-09	glycosylphosphatidylinositol-specific phospholipase C
KS17gene_372a	9.06	5.29E-05	lncRNA, putative
Tb927.4.4470	9.05	3.86E-09	adenylyl cyclase
KS17gene_3091a	9.04	1.86E-02	lncRNA, putative
KS17gene_371a	9.00	3.03E-03	lncRNA, putative
nt_1759.1	8.88	3.77E-04	lncRNA, putative
Tb927.6.3550	8.88	3.60E-03	phospholipid-translocating P-type ATPase (flippase), putative
Tb927.2.2015	8.80	6.93E-06	hypothetical protein
Tb927.11.15855	8.77	9.78E-09	hypothetical protein
Tb927.2.1160	8.77	3.56E-04	retrotransposon hot spot protein 2 (RHS2), interrupted, degenerate
Tb927.10.8480	8.68	8.11E-14	glucose transporter, putative
Tb927.2.1200	8.59	3.25E-09	DNA-directed RNA polymerase III subunit 2, pseudogene, putative
nt_445.1	8.57	2.40E-09	lncRNA, putative
Tb927.10.1040	8.52	1.08E-13	serine peptidase, Clan SC, Family S10
Tb927.4.4440	8.44	1.91E-10	adenylyl cyclase
nt_256.1	8.23	7.29E-04	lncRNA, putative
Tb927.8.8030	8.21	1.89E-11	emp24/gp25L/p24 family/GOLD, putative
nt_430.1	8.20	2.21E-04	lncRNA, putative
KS17gene_4362a	8.19	4.23E-03	lncRNA, putative
Tb927.7.2030	8.17	5.85E-03	retrotransposon hot spot protein 7 (RHS7), putative
Tb927.10.2400	8.15	1.85E-05	hypothetical protein
Tb927.3.5660	8.13	1.18E-06	UDP-GlcNAc:alpha3-D-mannoside beta-1,2-N-acetylglucosaminyltransferase I
Tb927.2.1250	7.98	5.14E-06	hypothetical protein
Tb927.7.3830	7.97	2.41E-05	kinesin K39, putative
KS17gene_1169a	7.96	6.44E-03	lncRNA, putative
Tb927.8.7900	7.96	5.57E-03	receptor-type adenylate cyclase GRESAG 4, putative
Tb927.5.350	7.84	2.18E-04	75 kDa invariant surface glycoprotein, putative
Tb927.2.950	7.82	1.64E-04	paraflagellar rod component, putative

Tb927.8.6720	7.80	4.06E-08	hypothetical protein, conserved
KS17gene_1443a	7.79	5.28E-03	lncRNA, putative
Tb927.1.350	7.79	1.63E-08	retrotransposon hot spot protein (RHS, pseudogene), putative
Tb927.11.12140	7.77	6.45E-03	hypothetical protein
Tb927.10.6720	7.76	7.05E-09	Plasma-membrane choline transporter, putative
Tb927.2.270	7.73	6.64E-03	retrotransposon hot spot protein 3 (RHS3), frameshift
KS17gene_4607a	7.72	1.56E-04	lncRNA, putative
Tb927.4.3380	7.71	1.70E-03	myosin IB heavy chain, putative
Tb927.9.15660	7.62	8.40E-08	procyclic-enriched flagellar receptor adenylate cyclase 6
Tb1.NT.24	7.60	3.68E-07	lncRNA, putative
KS17gene_3141a	7.59	4.10E-04	lncRNA, putative
KS17gene_4605a	7.55	1.59E-07	lncRNA, putative
KS17gene_1328a	7.54	2.26E-09	lncRNA, putative
Tb927.10.1050	7.52	2.22E-08	serine peptidase, Clan SC, Family S10
Tb927.11.6120	7.51	7.57E-03	ABC transporter, putative
Tb927.11.17840	7.45	1.69E-03	retrotransposon hot spot protein (RHS), degenerate
Tb927.6.5160	7.44	8.47E-06	retrotransposon hot spot protein 3 (RHS3), point mutation
Tb927.6.360	7.40	3.67E-04	UDP-Gal or UDP-GlcNAc-dependent glycosyltransferase (pseudogene), putative
KS17gene_2695a	7.35	3.93E-03	lncRNA, putative
Tb07.30D13.130	7.28	8.06E-06	hypothetical protein, conserved (pseudogene)
Tb927.10.6420	7.27	1.07E-02	hypothetical protein
Tb927.2.1310	7.26	4.91E-04	leucine-rich repeat protein (LRRP, pseudogene), putative
Tb927.2.1120	7.25	7.20E-06	retrotransposon hot spot protein 4 (RHS4), point mutation
Tb927.11.15850	7.25	2.00E-09	kinteoplast poly(A) polymerase complex 1 subunit
Tb927.1.480	7.09	2.20E-05	leucine-rich repeat protein (LRRP), putative
Tb927.7.1130	7.08	1.87E-11	trypanothione/tryparedoxin dependent peroxidase 2
Tb927.9.18140	7.01	3.91E-10	variant surface glycoprotein (VSG, pseudogene), putative
Tb927.2.300	6.98	4.22E-10	DNA-directed RNA polymerase, pseudogene, putative
Tb927.9.13070	6.96	8.02E-15	Heat shock factor binding 1 domain-containing protein
Tb927.10.6740	6.92	6.19E-11	Plasma-membrane choline transporter, putative
KS17gene_3751a	6.88	2.18E-05	lncRNA, putative
KS17gene_3345a	6.83	4.05E-03	lncRNA, putative
Tb927.1.60	6.79	1.14E-12	RNA polymerase (pseudogene), putative
KS17gene_5972a	6.78	3.56E-03	lncRNA, putative
Tb927.11.11600	6.73	1.74E-04	hypothetical protein, conserved
Tb927.11.1520	6.72	1.75E-04	expression site-associated gene 3 (ESAG3) protein, putative
Tb927.6.440	6.71	7.06E-07	haptoglobin-hemoglobin receptor
Tb927.5.630	6.61	2.64E-13	acidic phosphatase, putative
Tb927.2.5330	6.59	4.84E-03	hypothetical protein, conserved
KS17gene_4164a	6.56	6.01E-08	lncRNA, putative
Tb927.6.160	6.56	3.09E-08	retrotransposon hot spot protein 1 (RHS1), putative
Tb927.8.6730	6.48	1.07E-08	Enriched in surface-labeled proteome protein 24
Tb927_08_v4.snoRNA.0044	6.48	3.95E-06	H/ACA-like snoRNA
Tb927.11.15870	6.47	2.08E-09	hypothetical protein, conserved
Tb927.6.3470	6.45	1.00E-04	hypothetical protein, conserved
Tb927.2.460	6.42	3.70E-10	DNA-directed RNA polymerase, pseudogene, putative
Tb927.11.4770	6.38	1.79E-09	retrotransposon hot spot protein (RHS, pseudogene), putative
KS17gene_4419a	6.35	2.63E-11	lncRNA, putative
nt_451.3	6.34	2.03E-07	lncRNA, putative
Tb927.9.13650	6.31	1.32E-12	ADP-ribosylation factor, putative
Tb927.4.100	6.28	1.34E-10	retrotransposon hot spot protein 1 (RHS1), interrupted
Tb927.1.70	6.25	1.69E-02	retrotransposon hot spot protein 4 (RHS4), putative
Tb927.7.300	6.22	1.18E-10	UDP-Gal or UDP-GlcNAc-dependent glycosyltransferase, putative
Tb927.9.15930	6.22	9.30E-08	small GTPase, putative
Tb927.4.180	6.16	4.87E-07	hypothetical protein
KS17gene_1185a	6.15	1.13E-04	lncRNA, putative
KS17gene_6076a	6.11	1.13E-07	lncRNA, putative
Tb927.10.16190	6.09	8.28E-06	procyclic-enriched flagellar receptor adenylate cyclase 2
KS17gene_6855a	6.08	2.41E-03	lncRNA, putative
KS17gene_3712a	6.06	1.96E-04	lncRNA, putative
Tb11.NT.27	6.02	9.12E-04	lncRNA, putative
Tb927.10.70	6.01	3.93E-03	retrotransposon hot spot (RHS), putative, (fragment)

KS17gene_6769a	5.87	2.14E-03	lncRNA, putative
Tb927.11.5910	5.83	1.95E-05	WASH complex subunit 7, N-terminal/WASH complex subunit 7/WASH complex subunit 7, C-terminal, putative
Tb927.1.380	5.73	1.80E-08	Protein of unknown function (DUF1181), putative
KS17gene_2586a	5.71	2.52E-03	lncRNA, putative
KS17gene_7754a	5.70	2.56E-03	lncRNA, putative
Tb927.9.8950	5.70	1.41E-11	metallo- peptidase, Clan M- Family M48
Tb927.9.11480	5.69	6.56E-11	Enriched in surface-labeled proteome protein 9
Tb927.11.5210	5.66	1.77E-03	hypothetical protein, conserved
Tb927.10.3970	5.65	4.80E-12	hypothetical protein, conserved
Tb927.7.6590	5.64	1.26E-04	hypothetical protein, conserved
Tb927.10.14160	5.64	6.43E-10	Aquaglyceroporin 3
Tb927.3.5560	5.64	3.15E-06	hypothetical protein, conserved
Tb927.10.14140	5.63	1.24E-13	pyruvate kinase 1
Tb927.1.120	5.59	2.03E-13	retrotransposon hot spot protein 4 (RHS4), putative
Tb927.7.470	5.51	2.57E-07	Enriched in surface-labeled proteome protein 14
Tb927.10.8230	5.51	1.69E-13	protein disulfide isomerase 2
KS17gene_8043a	5.49	1.28E-02	lncRNA, putative
Tb927.4.4580	5.46	1.68E-03	hypothetical protein, conserved
Tb927.9.13380	5.45	8.10E-10	Autophagy-related protein 24
Tb927.7.6490	5.44	2.59E-06	hypothetical protein, conserved
Tb927.2.510	5.43	4.96E-04	retrotransposon hot spot protein 4 (RHS4), putative
Tb927.6.300	5.42	1.18E-02	receptor-type adenylate cyclase GRESAG 4, putative
Tb927.8.3900	5.41	1.34E-07	hypothetical protein, conserved
KS17gene_4037a	5.39	3.82E-05	lncRNA, putative
Tb927_08_v4.snoRNA.0043	5.38	6.19E-03	H/ACA-like snoRNA
KS17gene_2098a	5.37	2.03E-04	lncRNA, putative
Tb927.1.3670	5.36	7.41E-05	expression site-associated gene 8 (ESAG8) protein, putative
Tb927.2.5350	5.34	3.66E-03	hypothetical protein, conserved
KS17gene_846a	5.33	1.34E-02	lncRNA, putative
Tb927.2.170	5.32	1.62E-11	leucine-rich repeat protein 1 (LRRP1), putative
Tb927.11.11965	5.31	3.37E-12	cytoskeleton-associated protein, putative
Tb927.2.1080	5.30	1.64E-11	retrotransposon hot spot protein 5 (RHS5), putative
KS17gene_5447a	5.27	1.70E-03	lncRNA, putative
Tb927.4.2070	5.23	8.14E-07	antigenic protein, putative
Tb927.2.900	5.16	2.42E-08	hypothetical protein
Tb927.10.9710	5.15	1.99E-03	hypothetical protein
KS17gene_3132a	5.14	1.40E-02	lncRNA, putative
Tb927.7.1120	5.14	6.57E-10	trypanothione/tryparedoxin dependent peroxidase 1, cytosolic
Tb927.11.15490	5.08	2.89E-05	Tb-291 membrane associated protein, putative
Tb927.7.6860	5.08	3.00E-14	expression site-associated gene 5 (ESAG5) protein, putative
nt_17.3	5.06	2.70E-04	lncRNA, putative
Tb927.4.3980	5.01	1.02E-06	chaperone protein DnaJ, putative
nt_6689.1	5.00	7.38E-03	lncRNA, putative
KS17gene_6292a	4.96	2.17E-02	lncRNA, putative
Tb927.11.11980	4.96	4.14E-12	cytoskeleton-associated protein 15
Tb927.2.380	4.95	2.28E-07	retrotransposon hot spot protein 2 (RHS2), putative
Tb927.2.160	4.91	5.86E-03	Protein of unknown function (DUF1181), putative
KS17gene_5090a	4.90	3.26E-07	lncRNA, putative
Tb927.7.6070	4.90	1.04E-04	receptor-type adenylate cyclase GRESAG 4, putative
KS17gene_3810a	4.90	5.80E-04	lncRNA, putative
Tb927.10.8450	4.90	2.22E-02	glucose transporter 1E
KS17gene_7369a	4.89	9.04E-08	lncRNA, putative
Tb927.2.560	4.88	5.22E-10	retrotransposon hot spot protein 4 (RHS4), putative
KS17gene_4365a	4.85	6.01E-03	lncRNA, putative
KS17gene_329a	4.83	3.52E-03	lncRNA, putative
Tb927.9.2520	4.82	6.77E-07	microtubule-associated protein
Tb927.10.4180	4.81	6.82E-11	TFIIF-stimulated CTD phosphatase, putative
KS17gene_6325a	4.76	1.84E-08	lncRNA, putative
Tb927.6.560	4.75	7.87E-12	cysteine peptidase C (CPC)
Tb927.5.610	4.74	7.44E-10	acidic phosphatase, putative
Tb927.10.1770	4.73	1.02E-04	hypothetical protein

Tb927.6.130	4.73	1.54E-05	hypothetical leucine-rich repeat protein 1 (LRRP1)
Tb927.10.10490	4.70	5.47E-12	histone H2B, putative
Tb927.11.7080	4.69	5.31E-09	acidocalcisomal pyrophosphatase
Tb927.9.15700	4.68	2.35E-02	variant surface glycoprotein (VSG)-related, putative
Tb927.2.1344	4.68	2.10E-02	retrotransposon hot spot protein 1 (RHS1), interrupted
KS17gene_4884a	4.67	4.43E-03	lncRNA, putative
Tb927.7.3840	4.66	2.43E-02	kinesin-like protein, fragment, putative
KS17gene_5952a	4.66	4.56E-06	lncRNA, putative
Tb927.5.292b	4.64	1.48E-03	hypothetical protein
Tb927.11.1230	4.59	7.98E-05	hypothetical protein, conserved
Tb927.3.5180	4.59	1.72E-11	ADF/Cofilin
Tb927.5.840	4.56	4.52E-03	Nucleolar protein 111
Tb927.11.13040	4.54	1.14E-06	calmodulin
Tb927.7.4260	4.50	3.12E-09	Enriched in surface-labeled proteome protein 13
KS17gene_4614a	4.48	3.63E-08	lncRNA, putative
Tb927.3.5780	4.47	3.25E-05	hypothetical protein
KS17gene_7740a	4.45	9.88E-03	lncRNA, putative
Tb927.1.4970	4.44	1.11E-02	hypothetical protein
Tb927.6.150	4.42	2.13E-03	retrotransposon hot spot protein 3 (RHS3), frameshift
Tb927.2.6150	4.40	2.05E-04	adenosine transporter 2
nt_255.1	4.39	5.18E-03	lncRNA, putative
Tb6.NT.40	4.36	1.11E-03	lncRNA, putative
Tb927.6.3940	4.36	1.85E-07	Autophagy-related protein 27, putative
KS17gene_5976a	4.35	2.93E-05	lncRNA, putative
Tb927.11.11320	4.34	9.26E-05	hypothetical protein, conserved
Tb927.8.2260	4.34	3.01E-07	Present in the outer mitochondrial membrane proteome 39-2
Tb927.5.4590	4.29	2.96E-03	small GTP-binding rab protein, pseudogene, putative
Tb927.5.2410	4.29	1.01E-05	kinesin, putative
Tb927.8.5100	4.28	1.07E-03	hypothetical protein, conserved
Tb927.8.3890	4.27	5.95E-08	hypothetical protein, conserved
Tb927.11.12800	4.27	1.08E-05	ribonucleoside-diphosphate reductase small chain
KS17gene_213a	4.26	3.32E-04	lncRNA, putative
nt_417.2	4.21	6.96E-05	lncRNA, putative
Tb927.7.2650	4.21	6.24E-10	Cytoskeleton associated protein 51V
Tb927.5.4380	4.20	7.96E-04	Kinetoplastid-specific Protein Phosphatase 1
Tb927.8.4060	4.19	5.37E-09	Flagellum adhesion protein 2, putative
Tb927.3.3450	4.16	1.51E-12	ADP-ribosylation factor-like protein 3, putative
Tb927.1.270	4.15	2.23E-05	hypothetical protein
Tb927.10.8350	4.13	1.87E-02	hypothetical protein
Tb927.7.480	4.13	3.12E-04	hypothetical protein, conserved
Tb927_09_v4.snoRNA.0027	4.11	7.02E-03	C/D snoRNA
Tb927.1.4710	4.11	1.65E-03	hypothetical protein, conserved
Tb927.1.3860	4.08	2.91E-04	hypothetical protein, conserved
KS17gene_4622a	4.06	1.94E-10	lncRNA, putative
Tb927.5.360	4.05	4.00E-12	75 kDa invariant surface glycoprotein
KS17gene_1897a	4.05	1.88E-04	lncRNA, putative
Tb927.9.6570	4.03	3.34E-03	hypothetical protein, conserved
Tb927.11.5370	4.02	9.19E-07	hypothetical protein, conserved
Tb927.4.1200	4.02	1.87E-03	expression site-associated gene 1 (ESAG1) protein, putative

Supplementary Table S7. Differential gene expression. Genes up-regulated in procyclic-stage trypanosomes.

**gene-ID**	**fold change (PC/BS)**	**adjusted *p*-value**	**gene product**
Tb927.10.10270	61.83	4.82E-24	hypothetical protein
Tb927.10.10260	44.01	7.95E-30	EP1 procyclin
Tb927.10.10250	33.52	2.17E-21	EP2 procyclin
KS17gene_6791a	32.87	1.50E-10	lncRNA, putative
Tb927.3.590	32.66	1.65E-17	adenosine transporter, putative
Tb927.6.450	28.44	7.21E-23	procyclin PARP A
Tb927.6.520	26.59	6.68E-19	EP3-2 procyclin
Tb927.5.2260	26.29	8.65E-24	conserved protein
nt_6401.1	22.24	9.88E-07	lncRNA, putative
Tb927.4.4000	22.15	9.28E-12	amino acid transporter, putative
Tb927.7.3020	20.76	7.21E-23	Nitroreductase family, putative
nt_3670.2	19.73	8.15E-06	lncRNA, putative
Tb927.7.2980	18.85	2.05E-23	Nitroreductase family, putative
Tb927.7.430	18.63	3.94E-07	hypothetical protein, conserved
Tb927.7.440	18.60	8.98E-07	hypothetical protein, conserved
Tb927.10.10240	18.21	1.78E-09	procyclin-associated gene 1 (PAG1) protein
Tb927.10.15410	16.90	6.09E-19	glycosomal malate dehydrogenase
Tb927.11.7500	16.77	1.75E-20	Protein of unknown function (DUF423), putative
Tb927.5.4020	16.41	2.72E-12	hypothetical protein
Tb927.6.3490	16.06	3.23E-16	zinc finger protein 1
Tb927.7.340	15.85	3.05E-05	Alpha/beta hydrolase family, putative
Tb927.6.480	15.55	8.03E-12	surface protein EP3-2 procyclin precursor
Tb927.5.990	14.80	5.74E-10	hypothetical protein, conserved
Tb927.10.8490	13.86	4.26E-19	glucose transporter, putative
Tb927.7.6883	13.77	3.64E-11	28S alpha ribosomal RNA
Tb927.11.6280	13.04	2.56E-16	pyruvate phosphate dikinase
Tb927.11.1710	12.57	2.17E-21	guide RNA-binding protein of 21 kDa
Tb927.11.2410	12.52	1.65E-17	Flabarin, putative
Tb927.2.4610	12.29	1.71E-18	branched-chain amino acid aminotransferase, putative
Tb927.7.6180	12.21	2.59E-04	hypothetical protein, conserved
Tb927.6.3880	12.13	4.11E-05	hypothetical protein, conserved
Tb927.8.480	12.00	1.44E-05	phosphatidic acid phosphatase protein, putative
Tb927.6.970	11.94	1.69E-16	cysteine peptidase, Clan CA, family C1, Cathepsin L-like
Tb927.9.15550	11.83	4.49E-06	BARP protein
Tb927.11.7490	11.64	2.84E-18	Protein of unknown function (DUF423), putative
Tb927.11.4700	11.37	2.17E-21	prostaglandin f synthase
Tb927.8.8300	10.88	4.80E-06	amino acid transporter, putative
Tb927.10.15090	10.73	1.77E-16	hypothetical protein, conserved
Tb927.1.710	10.57	2.61E-17	phosphoglycerate kinase
Tb927.8.2540	10.16	2.27E-18	3-ketoacyl-CoA thiolase
Tb927.3.3449	10.08	1.42E-05	28S alpha ribosomal RNA
Tb927.10.9080	9.85	7.23E-14	pteridine transporter, putative
Tb927.11.3730	9.82	1.08E-06	leucyl-tRNA synthetase, putative
Tb927.7.2210	9.75	4.89E-05	hypothetical protein, conserved
Tb927.6.1010	9.69	9.81E-15	cysteine peptidase, Clan CA, family C1, Cathepsin L-like
Tb927.10.4280	9.47	4.44E-15	ubiquinol-cytochrome c reductase complex 14 kda protein
Tb927.11.9980	9.37	3.01E-07	2-oxoglutarate dehydrogenase E1 component, putative
Tb927.9.7470	9.28	9.60E-10	purine nucleoside transporter
Tb927.10.13430	9.20	2.03E-05	citrate synthase, putative
Tb927.7.5970	8.62	4.98E-16	protein associated with differentiation 5, putative
Tb927.11.5440	8.46	1.45E-12	NADP-dependent malic enzyme, cytosolic
Tb927.10.8750	8.39	5.98E-04	GTPase activating protein, putative
Tb927.6.500	8.31	6.12E-10	gene related to expression site-associated gene 2 (GRESAG2), putative
Tb927.2.4210	7.98	1.13E-17	Phosphoenolpyruvate carboxykinase [ATP], glycosomal
Tb927.10.15900	7.85	8.43E-09	hypothetical protein, conserved
Tb927.11.8990	7.79	8.27E-08	cation transporter, putative
Tb927.11.15550	7.75	3.93E-08	NADH-cytochrome b5 reductase, putative
Tb927.8.6750	7.68	1.09E-13	translationally controlled tumor protein (TCTP), putative
Tb927.8.1550	7.66	1.77E-03	paraflagellar rod component, putative
Tb927.10.2190	7.54	9.99E-04	Protein of unknown function (DUF667), putative
Tb927.5.2160	7.48	7.46E-12	conserved protein
Tb927.10.3210	7.43	1.34E-12	delta-1-pyrroline-5-carboxylate dehydrogenase, putative

Tb927.10.2560	7.13	4.82E-15	mitochondrial malate dehydrogenase
Tb927.10.9350	7.12	1.06E-04	hypothetical protein, conserved
Tb927.10.5760	7.10	1.29E-04	adenylate kinase, putative
Tb927.6.530	7.09	1.17E-11	procyclin associated gene 3 (PAG3) protein
Tb927.10.8500	7.05	1.42E-10	glucose transporter, putative
Tb927.11.3610	6.96	2.43E-13	nucleobase/nucleoside transporter 8.1
Tb927.3.1840	6.95	4.09E-06	3-oxo-5-alpha-steroid 4-dehydrogenase, putative
Tb927.6.760	6.94	2.02E-05	receptor-type adenylate cyclase GRESAG 4, putative
Tb927.11.11360	6.90	9.22E-20	receptor for activated C kinase 1
Tb927.5.2560	6.74	2.73E-12	hypothetical protein, conserved
Tb11.02.5400	6.70	1.17E-11	cystathionine beta-synthase, putative
Tb927.7.4070	6.68	1.62E-14	cysteine peptidase, Clan CA, family C2, putative
Tb927.3.3432	6.59	3.79E-06	28S alpha ribosomal RNA
Tb927.11.rRNA_1	6.56	1.45E-12	5.8S ribosomal RNA
Tb927.1.3800	6.51	8.41E-04	Present in the outer mitochondrial membrane proteome 18
Tb927.10.2350	6.48	2.24E-06	pyruvate dehydrogenase complex E3 binding protein, putative
Tb927.4.3950	6.35	4.35E-09	cytoskeleton-associated protein CAP5.5, putative
Tb927.3.3441	6.35	1.47E-05	28S alpha ribosomal RNA
Tb927.10.12780	6.32	9.89E-07	Zinc finger CCCH domain-containing protein 37
Tb927.1.2210	6.26	2.61E-07	nucleosome assembly protein (NAP), putative
Tb927.1.4630	6.18	3.58E-03	cyclin-like F-box protein 1E
Tb927.9.7920	6.14	2.31E-03	hypothetical protein, conserved
Tb927.9.15540	6.04	3.11E-08	BARP protein
Tb927.7.1320	5.97	2.40E-17	10 kDa heat shock protein, putative
Tb927.10.4030	5.95	1.14E-03	hypothetical protein
Tb927.4.1360	5.92	1.97E-09	Glucose-6-phosphate 1-epimerase, putative
Tb927.6.2070	5.92	5.85E-05	Mitoribosomal SSU assembly factor 28
Tb927.6.200	5.91	3.24E-06	receptor-type adenylate cyclase GRESAG 4, putative
Tb927.8.6170	5.87	1.86E-12	transketolase, putative
Tb927.10.10770	5.86	2.20E-11	Generative cell specific 1 protein, putative
Tb927.10.8530	5.84	1.36E-10	glucose transporter 2A
Tb927.10.6860	5.83	5.04E-04	endonuclease v
Tb927.2.1443	5.75	1.64E-12	5.8S ribosomal RNA
Tb927.11.4760	5.72	1.30E-07	hypothetical protein
Tb927.7.4390	5.70	1.87E-12	threonine synthase, putative
Tb927.9.6760	5.62	2.27E-03	hypothetical protein, conserved
Tb927.10.470	5.51	5.43E-09	choline dehydrogenase, putative
Tb927.8.4720	5.49	1.16E-12	amino acid transporter, putative
Tb927.8.7670	5.42	3.68E-09	amino acid transporter, putative
Tb927.1.2820	5.35	8.34E-13	pteridine transporter, putative
Tb927.11.3270	5.34	3.42E-04	squalene monooxygenase, putative
Tb927.10.11220	5.32	7.51E-14	procyclic form surface phosphoprotein
Tb927.2.3920	5.31	4.84E-08	Complex 1 protein (LYR family), putative
Tb927.7.5400	5.28	4.06E-05	hypothetical protein, conserved
Tb927.11.16730	5.25	2.02E-08	dihydrolipoyl dehydrogenase
Tb927.11.12490	5.25	1.86E-03	hypothetical protein, conserved
Tb927.6.4790	5.24	1.89E-10	hypothetical protein, conserved
Tb927.9.1520	5.22	1.12E-13	hypothetical protein, conserved
Tb927.1.2880	5.20	3.53E-11	pteridine transporter, putative
Tb927.3.3890	5.10	1.26E-05	hypothetical protein, conserved
Tb927.7.6850	5.08	2.19E-08	trans-sialidase
Tb927.1.2470	5.03	9.81E-15	histone H3, putative
KS17gene_1079a	5.00	1.01E-09	lncRNA, putative
Tb927.9.2320	4.96	2.14E-05	methyltransferase domain containing protein, putative
Tb927.11.9750	4.94	1.08E-06	Protein of unknown function (DUF498/DUF598), putative
Tb927.11.14000	4.94	3.94E-16	nuclear RNA binding domain 1
Tb927.7.5990	4.94	8.54E-08	protein associated with differentiation 7, putative
Tb927.6.610	4.91	3.27E-04	kinetoplast ribosomal PPR-repeat containing protein 18
Tb927.10.10000	4.91	8.34E-13	hypothetical protein, conserved
Tb927.9.7980	4.91	1.45E-12	hypothetical protein, conserved
Tb927.9.2450	4.88	2.60E-03	electon transport protein SCO1/SCO2, putative
Tb927.4.4620	4.80	4.94E-13	cytochrome oxidase subunit VIII
Tb927.10.690	4.78	4.20E-06	palmitoyl acyltransferase 3, putative
Tb927.2.1953	4.77	2.73E-06	28S alpha ribosomal RNA
Tb927.9.15580	4.73	8.06E-09	BARP protein
Tb927.10.6200	4.72	9.04E-09	hypothetical protein, conserved
Tb927.6.3800	4.69	1.73E-08	heat shock 70 kDa protein, mitochondrial precursor, putative
Tb927.1.2400	4.67	2.68E-16	alpha tubulin
Tb927.10.15950	4.63	2.93E-05	TATA-box-binding protein

Tb927.3.700	4.60	4.16E-09	hypothetical protein, conserved
Tb927.5.3890	4.59	8.09E-06	hypothetical protein, conserved
Tb927.8.1640	4.59	2.05E-13	MSP-B, putative
Tb927.3.3750	4.59	3.26E-07	paraflagellar rod component, putative
Tb927.8.4010	4.53	2.24E-06	Flagellum adhesion protein 1
Tb927.5.440	4.50	1.06E-09	trans-sialidase, putative
Tb927.11.7260	4.48	3.06E-07	hypothetical protein, conserved
Tb927.6.5095	4.41	1.99E-12	hypothetical protein
Tb927.11.12440	4.40	8.29E-05	Plus-3 domain/Zinc finger, C3HC4 type (RING finger), putative
Tb927.4.3990	4.39	1.74E-04	amino acid transporter, putative
Tb927.5.1060	4.38	1.95E-09	mitochondrial processing peptidase, beta subunit, putative
Tb927.3.3423	4.35	5.71E-05	28S alpha ribosomal RNA
Tb927.7.5980	4.35	5.79E-04	protein associated with differentiation 6, putative
Tb927.11.1800	4.35	3.91E-14	histone H1
Tb927.9.5890	4.33	1.75E-11	solanesyl-diphosphate synthase, putative
Tb927.7.1790	4.23	1.62E-07	Adenine phosphoribosyltransferase, putative
Tb927.9.7620	4.14	5.51E-16	60S ribosomal protein L11, putative
Tb927.9.14160	4.13	9.43E-08	rieske iron-sulfur protein, mitochondrial precursor
Tb927.2.3030	4.10	8.64E-05	ATP-dependent Clp protease subunit, heat shock protein 78 (HSP78), putative
Tb927.3.2880	4.10	2.29E-09	Mitochondrial ATP synthase subunit, putative
Tb927.5.930	4.07	1.20E-08	NADH-dependent fumarate reductase
Tb927.11.13140	4.03	2.27E-12	cytochrome oxidase subunit X
Tb927.3.3431	4.01	5.63E-11	5.8S ribosomal RNA
Tb927.9.7830	4.00	1.03E-11	tRNA import complex component, putative
